# Next—Generation Diagnostic Technologies for Dengue Virus Detection: Microfluidics, Biosensing, CRISPR, and AI Approaches

**DOI:** 10.3390/s26010145

**Published:** 2025-12-25

**Authors:** Salim El Kabbani, Gameel Saleh

**Affiliations:** Department of Biomedical Engineering, College of Engineering, Imam Abdulrahman Bin Faisal University, P.O. Box 1982, Dammam 31441, Saudi Arabia

**Keywords:** dengue virus detection, biosensor, optical biosensors (SPR; LSPR; SERS), electrochemical biosensors (DPV; EIS), NS1 antigen, microfluidic lab–on–chip, CRISPR diagnostics, artificial intelligence in viral detection

## Abstract

Dengue fever remains a major mosquito–borne disease worldwide, with over 400 million infections annually and a high risk of severe complications such as dengue hemorrhagic fever. The disease is prevalent in tropical and subtropical regions, where population density and limited vector control accelerate transmission, making early and reliable diagnosis essential for outbreak prevention and disease management. Conventional diagnostic methods, including virus isolation, reverse transcription polymerase chain reaction (RT–PCR), enzyme–linked immunosorbent assays (ELISA), and serological testing, are accurate but often constrained by high cost, labor–intensive procedures, centralized laboratory requirements, and delayed turnaround times. This review examines current dengue diagnostic technologies by outlining their working principles, performance characteristics, and practical limitations, with emphasis on key target analytes such as viral RNA; nonstructural protein 1 (NS1), including DENV–2 NS1; and host antibodies. Diagnostic approaches across commonly used biofluids, including whole blood, serum, plasma, and urine, are discussed. Recent advances in biosensing technologies are reviewed, including optical, electrochemical, microwave, microfluidic, and CRISPR–based platforms, along with the integration of artificial intelligence for data analysis and diagnostic enhancement. Overall, this review highlights the need for accurate, scalable, and field–deployable diagnostic solutions to support early dengue detection and reduce the global disease burden.

## 1. Introduction

Dengue is an arbovirus that is mainly transmitted by mosquitoes. It is among the most dangerous re–emerging diseases in humans. Dengue is the primary arboviral cause of morbidity and mortality in tropical and subtropical regions. Dengue is a flu–like infection that may develop into a fatal or severe illness, such as dengue shock syndrome or hemorrhagic fever. The spread of the dengue virus (DENV) has increased exponentially over the last five decades and has expanded from urban to rural areas in the last decade. Despite ongoing vector control efforts and the widespread distribution of clinical guidelines, this rate continues to rise. According to the World Health Organization (WHO), up to 400 million dengue infections are estimated annually. Early detection during outbreaks plays a critical role in effective disease containment and reduction in disease spread [1].

Several factors influence the transmission rate of the disease. High population density, poor hygienic conditions, degradation of public services, and weak vector control measures are major contributors to outbreaks, along with rapid urbanization and increased traffic conditions [2]. Temperature also plays a significant role in the spread of DENV, as the transmission rate increases with higher temperatures. In areas with favorable weather, poverty and limited control measures are the main aspects of disease emergence [3].

In 2023, dengue fever reached its highest recorded outbreak to date, with Brazil reporting 3.9 million cases. A total of 6.5 million cases and more than 6800 deaths have been reported in 80 countries. The number of cases has increased to 13 million, with more than 8500 dengue–related deaths in 2024. These numbers are estimated to increase exponentially in the coming years, underscoring the need for a global approach to control the spread and infection of DENV. Dengue fever is spreading at an alarming rate due to climate change and increased urbanization. In conflict–affected countries such as Sudan and Afghanistan, mass refugee movements due to economic and war crises have introduced dengue fever to new areas. Natural disasters, such as floods, contribute to the spread of dengue by contaminating and polluting water supplies, creating ideal habitats for dengue–carrying mosquitoes. In countries such as Oman and Saudi Arabia, the sudden increase in rainfall and massive population movement have caused a surge in reported cases [4,5].

In recent years, Saudi Arabia has experienced a rise in dengue fever incidence, particularly in densely populated urban regions. From the late 2022 to early 2023, a total of 9729 cases were reported over a 31-week period, with most cases concentrated in central and eastern Jeddah [6]. During the first half of 2023 alone, 4099 cases were recorded [7]. An awareness campaign conducted in Jeddah in 2024 indicates that dengue remains an ongoing public health concern [8]. Saudi Arabia is geographically connected to neighboring countries such as Oman, Yemen, and Sudan, where recurrent dengue outbreaks have also been reported. The high concentration of cases in urban settings, together with seasonal rainfall and large–scale population movement, places increased pressure on laboratory testing. This highlights the need for next–generation diagnostic technologies that enable rapid, sensitive, and point–of–care detection, allowing early case identification, timely clinical decision–making, and improved outbreak surveillance in high–risk regions.

Owing to climate change, Aedes aegypti mosquitoes, which are the primary carriers of arboviruses, including dengue, have migrated to many countries. Currently, Aedes aegypti has been confirmed in 164 countries [9]. This underscores the need to strengthen vector and disease control measures to prevent a worldwide dengue outbreak, which could lead to severe economic burdens and global quarantines, disrupting everyday life in much the same way as the COVID–19 pandemic. Many countries are still recovering from the COVID–19 pandemic, and additional large outbreaks could strain public health systems and communities. Public awareness is a principal measure in the management of DENV. Preventive measures are the first line of defense against the spread of DENV–carrying mosquitoes. Contact with mosquitoes in DENV outbreak regions can be reduced using various techniques, such as insecticide–covered nets, mosquito repellents, wearing protective clothes (such as gloves), and managing water and waste [10]. Currently, research institutes are racing to create a vaccine, but only CYD–TDV has been FDA–Approved [11].

**Figure 1:**
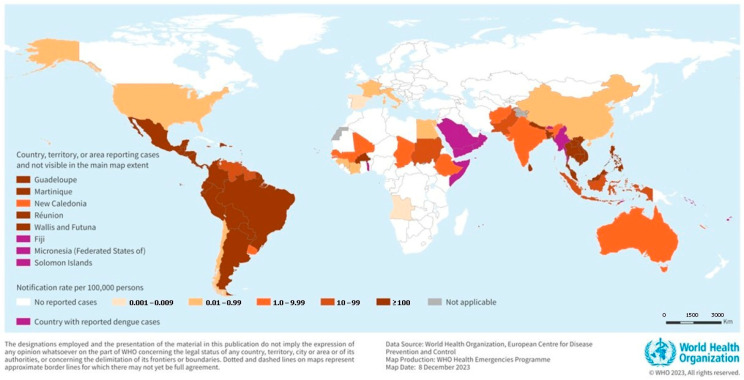
The global burden and distribution of Dengue in 2023. Reprinted with permission from Ref. [4]. Copyright 2023 World Health Organization (WHO).

The primary host of DENV is humans. Female mosquitoes feed on the blood of infected individuals. The virus enters the mosquito midgut and travels through the body for eight to twelve days. Mosquitoes carrying the virus are infectious for the remainder of their lives. The virus is transmitted when a mosquito feeds on the blood of a healthy individual. Weather conditions also affect the virus incubation period [12]. Aedes aegypti and Aedes albopictus mosquitoes are among the most effective arbovirus carriers because they prefer to live around people and bite multiple times. Aedes aegypti are species usually found in Africa that breed in forests. It is a feral species that is native to Africa and commonly occurs in the presence of humans. During winter, the species disappear from areas above and below the 10 °C isotherms in the northern and southern latitudes [13], decreasing the viral transmission rate. This indicates that temperature significantly affects dengue transmission, as shown in Figure 1.

DENV belongs to the Flavivirus family, which contains more than 70 viruses of the genus. DENV has four antigenically dissimilar but related serotypes: DENV–1, DENV–2, DENV–3, and DENV–4; a fifth serotype, DENV–5, was reported in 2013 [14]. The origin of DENV is not entirely known, but recent research on dengue and other flaviviruses proposes that they may have originated in Africa, and another study suggests that they may have originated in Asia. All four dengue serotypes have been documented in Asia [15], whereas only DENV–2 has been reported in Africa [16]. Dengue infection is often diagnosed based on fever–like symptoms. However, this approach is limited because d can be confused with other febrile illnesses, complicating early diagnosis efforts.

## 2. Physiology

DENV is a single–stranded positive–sense RNA molecule flavivirus belonging to the Flaviviridae family. RNA viruses are completely made up of RNA and do not need a DNA intermediate in their viral life cycle; therefore, they can be categorized as positive–sense or negative–sense viruses [17]. The Flaviviridae family accommodates three genera, Flavivirus, Pestivirus, and Heptacavirus. Genus Flavivirus includes arboviruses such as dengue virus, West Nile virus, yellow fever virus, and encephalitis viruses.

**Figure 2:**
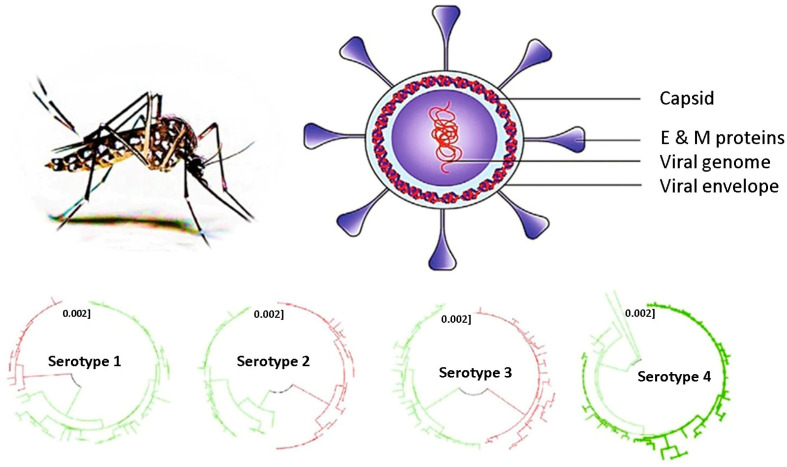
Dengue virus physiology and its four serotypes. Different colors are used to distinguish phylogenic lineages within each DENV serotype. Reprinted with permission from Ref. [19].

DENV comprises a genome of 11,000 nucleotides, a diameter of 40–50 nm and has a single open reading frame that encodes a polyprotein precursor of sequence NH2–C–prM–E–NS1–NS2A-NS2B-NS3-NS4A-NS4B-NS5-COOH. The viral genomic RNA is encapsulated by the C protein, forming the nucleocapsid. The viral membrane (prM protein) and envelope protein (E protein) are embedded in the lipid bilayer surrounding the nucleocapsid. These three structural proteins create the entire infectious virus particle (virion). The non-structural proteins (NS1–NS5) are crucial for virion assembly, viral replication, and evading the immune response [18], as shown in Figure 2.

DENV has four slightly antigenically distinct serotypes (DENV–1 to –4), each of which interacts differently with cell receptors and induces a distinct immune response within the infected host. Due to different immune responses, immunity for a specific serotype does not grant immunity for other serotypes [20], so getting infected with a different serotype from the original infection may lead to severe sickness and complications.

These physiological features guide the development of diagnostic technologies for DENV. The NS1 protein is secreted into the bloodstream during the early stages of infection, making it a key target for antigen–based assays and biosensors. Antigenic epitopes on the E protein are utilized for antibody recognition and serotype–specific detection in immunoassays and plasmonic–based platforms. Additionally, the viral RNA genome serves as the target for nucleic acid–based technologies such as polymerase chain reaction (PCR) and CRISPR–based diagnostics, paving the way for highly sensitive early detection.

To provide context for the diagnostic techniques discussed in this review, Table 1 summarizes the key dengue analytes, their corresponding detection biofluids, and representative concentration ranges reported in clinical samples.

## 3. Dengue Detection Techniques and Recent Advances

DENV fever symptoms can range from asymptomatic to severe. A second infection with a different serotype may lead to dengue hemorrhagic fever, which can lead to internal bleeding, organ failure, and eventually death [21]. Early disease symptoms are flu/fever–like, making DENV challenging to diagnose and identify. The need for accessible, affordable, accurate, and rapid procedures for the early diagnosis of dengue fever is of utmost importance. DENV diagnosis is performed by isolating the virus in cell cultures, genomic detection, serological testing, and using biosensors. Conventional diagnostic methods such as cell culture, PCR, and enzyme–linked immunosorbent assays (ELISA) are reliable but are often limited by long processing times, laboratory–based requirements, and higher costs. Cell culture may take several days, while PCR and ELISA typically require hours. In contrast, emerging biosensing technologies demonstrate promising results and performance improvements in diagnostic procedures, achieving detection within minutes to under an hour, with limits of detection improving from ng/mL levels in ELISA to picogram or femtomolar ranges in electrochemical, optical, and CRISPR–based platforms, and from 10^2^–10^3^ viral copies per milliliter in PCR to only a few copies. This order–of–magnitude improvement highlights the potential of advanced diagnostic platforms for rapid and sensitive dengue detection. To address these constraints, recent research has focused on emerging diagnostic technologies that improve speed, sensitivity, and field deployment while maintaining analytical accuracy.

### 3.1. Cell Culture Isolation

One of the most prominent techniques for diagnosing viral infections is isolating them in cell cultures. Virus isolation can aid in the early diagnosis of emerging and re–emerging viruses. Infected host cells are cultured in a suitable growth medium to separate the virions from the cell culture using various methods. This approach yields large amounts of infectious viruses and can isolate and identify many viruses, making it a very effective diagnostic method. Treatments can be added to cell cultures to observe the response of viruses and determine whether the treatments are effective. Infected samples should be collected within 5–7 days after the onset of viral symptoms. DENV can be obtained from blood, serum, or plasma. Infected samples are usually cultured using a monolayer of Aedes albopictus clone larval cells (C6/36 cells) or the mosquito inoculation technique [22]. Mosquito inoculation is effective for isolating DENV; however, this method is difficult to implement because it requires good coordination, and most laboratories do not have access to insectaries [23]. Following isolation, Serological diagnosis is performed using immunofluorescence antibody testing with monoclonal antibodies [24].

Cell culture isolation is the main technique used to isolate DENV, study its physiology, and develop new sensors to ease the diagnostic process. The development of a rapid gold nanoparticle–based lateral flow immunoassay biosensor in 2022 successfully detected DENV after its isolation and propagation in C6/36 cell cultures [25]. Virus isolation provides large quantities of the virus, but the expertise needed to implement this technique, the time it consumes, and the expensive laboratory equipment needed must all be considered before carrying out this procedure.

### 3.2. Genomic Detection

**Figure 3:**
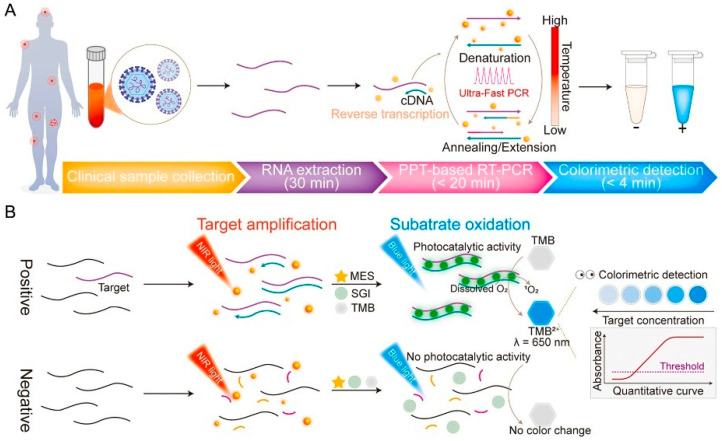
Workflow of the proposed plasmonic colorimetric RT–PCR sensor (PPT–RTcPCR) (**A**) and schematic illustration of the detection mechanism and colorimetric signal generation (**B**). Arrows represent the process flow, whereas colors and line styles are used to schematically differentiate assay stages and reaction components. Reprinted with permission from Ref. [30].

Genomic detection relies on identifying a virus either by its viral genome or by a part of its genomic sequence. Genomic detection of DENV is usually performed using reverse transcriptase–polymerase chain reaction (RT–PCR) or real–time quantitative RT–PCR (RT–qPCR). PCR is a Nucleic Acid Amplification Test (NAAT) technique that amplifies a specific part of the RNA sequence. This leads to a huge increase in the viral particle count and can make the identification of viruses easier. Quantitative PCR is a variation in the PCR in which the concentration of the amplified virus can be quantified. A fluorescent dye is usually used, and the fluorescence intensity is measured to determine the yield concentration [26]. The excellent sensitivity, specificity, and speed of nucleic acid detection make PCR one of the most sought–after techniques for DENV detection, with reported detection limits reaching as low as a few copies of RNA per reaction [27]. Since DENV is an RNA virus, a reverse transcriptase reaction must be performed. PCR is usually performed 3–5 days after the onset of symptoms; after the 5th day, the sensitivity of PCR decreases gradually [28]. PCR enables detection of dengue virus within a few hours and provides high analytical specificity for viral RNA. However, it lacks the ability to identify DENV serotypes. Despite its sensitivity, the need for expensive equipment, skilled laboratory personnel, and the risk of cross–contamination, which may cause false–positive results, should be noted. Therefore, strict quality control procedures must be followed by experienced technicians to avoid such risks [29]. The expensive equipment needed to perform this test is not available in economically burdened areas, where the prevalence of dengue is highest. Approaches integrating PCR with biosensors have led to significant advances in virus detection, a biosensor combining plasmonic RT–PCR with colorimetry was confirmed to have detected DENV RNA with specificity of 97.5% within 54 min, the workflow of this sensor is shown in Figure 3 [30]. Combining biosensors with existing diagnostic techniques to benefit from their combined advantages has recently become a popular point of interest among researchers.

### 3.3. Serological Testing

**Figure 4:**
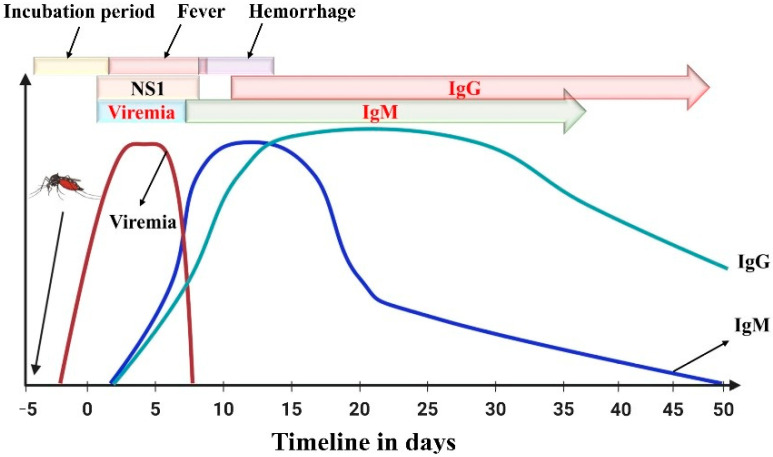
Immune response (IgM and IgG antibodies) following dengue infection. Day 0 corresponds to the onset of clinical symptoms, and negative numbers represent the pre–symptomatic phase. Reprinted with permission from Ref. [31].

Serological tests can detect the presence of specific antibodies in a sample. It is simple and inexpensive, making it a versatile diagnostic tool. Cerebrospinal and serum samples are used to test for antibodies, where specific antibodies indicate an existing infection. DENV, among other flaviviruses, can be diagnosed using antibodies released approximately 5 days after infection. Immunoglobulin antibody classes Ig(G, M and A) directed towards the envelope E protein of flavivirus antigens can be detected using this method. During the beginning of the infection, IgM antibodies are mainly produced and may remain in the body for up to 3 months. The IgM antibody response is higher in the first instance of dengue infection, whereas the IgG antibody response is more prevalent during secondary infections. IgG antibodies may remain in the body for years, as shown in Figure 4. The long periods of time during which IgG antibodies persist in the body can give false positives against flaviviruses [31,32]. The most commonly used assays to check for the presence of IgM and IgG antibodies during dengue infection are lateral flow strips, immunofluorescence–based assays, and ELISA.

**Figure 5:**
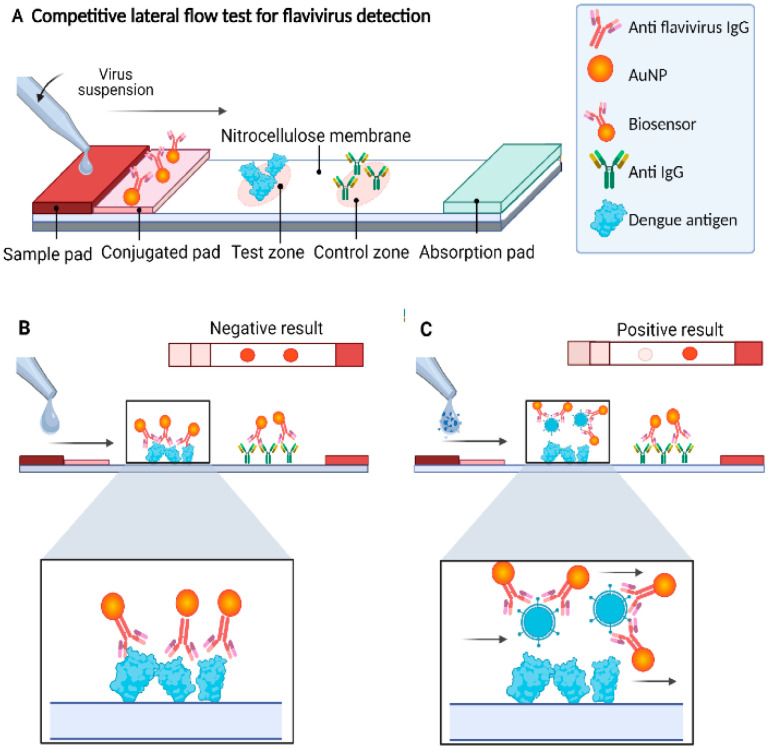
(**A**) Schematic of the AuNP–based lateral flow immunoassay sensor and (**B**) the negative result shows two reddish spots produced by AuNPs. (**C**) A positive result shows only one reddish spot. Arrows indicate the direction of sample flow and binding interactions, while colors are used to distinguish AuNPs, antigens, antibodies, and competitive binding outcomes. Reprinted with permission from Ref. [25].

Lateral Flow Assays (LFA) are paper–based assays that can quickly detect biomarkers (5–30 min). LFAs are inexpensive, rapid, small, portable, and simple to use, making them suitable for POC diagnosis, and they can be found in hospitals and laboratories. LFA–based tests use blood, serum, and plasma samples to determine the presence of analytes. A blood sample is applied onto the strip, it travels forward by capillary action. As it moves, the target analyte binds to mobile detection reagents, usually being gold nanoparticles (AuNPs) conjugated to detection antibodies. This complex continues toward the test line, where immobilized monoclonal capture antibodies are located. The accumulation of AuNPs at this line generates a visible reddish spot that indicates a positive result. A control line is placed further along the strip to verify that the sample has traveled correctly. LFAs combined with other techniques can improve the detection accuracy. The study in [25] proposed a biosensor that uses a combined LFA and AuNPs system to detect DENV using 4G2 antibodies (IgG clone). The designed biosensor successfully detected dengue antigens in 10 min with an estimated detection limit of 5.12 × 10^2^ PFU and has the potential to be used in POC tests. Figure 5 shows a schematic of the proposed sensor.

Immunofluorescence–based assays (IFA) detect viral antigens using antibodies labeled with fluorescent molecules. When these antibodies bind to their target, the fluorophore is excited by a specific light source and emits a detectable fluorescent signal. Signal intensity can be further enhanced using fluorescent secondary antibodies. IFAs are commonly used for identifying viral pathogens such as HIV and DENV. The generated fluorescence provides a direct visual confirmation of antigen presence and enables sensitive detection of viral biomarkers. Immunofluorescence testing can be coupled with ELISA to confirm the presence of the target antibodies. An immunofluorescence biosensor designed to detect the NS1 structural protein in infected blood plasma achieved an LOD of 15 ng/mL. The biosensor contains specific monoclonal NS1 antibodies, and antigen determination is performed using fluorescein isothiocyanate (FITC) conjugated to IgG antibodies, which provides low cross–reactivity with other flaviviruses, making it a promising tool for clinical applications [33].

ELISA detects viral antigens or antibodies by binding the target to an immobilized capture antibody, followed by attachment of an enzyme–linked detection antibody. The enzyme converts a chromogenic substrate into a measurable color signal. The sandwich ELISA technique produces a visible color change that can be detected using a microplate reader were the color intensity correlates directly with antigen concentration. This detection method is easy to perform, fast, and highly specific, making it a reference for immunoassay testing techniques. ELISA tests have many samples, such as blood, serum, plasma, urine, saliva, and cerebrospinal fluid. Advancements in the utilization of ELISA for viral detection have been made by designing new immunoassays by modifying the materials used. A sandwich ELISA using primary and secondary antibodies coupled with a thionicotinamide–adenine dinucleotide (thio–NAD) cycling signal amplification system achieved an LOD of 1.152 pg/mL and a sensitivity and specificity of 98.3% and 100%, respectively [34,35]. A comparison of the standard clinical approaches is presented in Table 2.

Despite their clinical reliability, conventional diagnostic methods are limited by long processing times, centralized laboratory requirements, and the need for skilled personnel, which restrict their applicability for early and point–of–care dengue diagnosis. These limitations have driven the development of biosensor–based platforms that enable faster, portable, and sensitive detection.

### 3.4. Biosensors

Biosensors detect biological reactions and convert the results into measurable signals [45]. Biological recognition elements can quantify a wide range of biomolecules in small amounts of biological samples. Biosensors are quick, reliable, and robust, making them a widely studied topic in biomedical research and development. DENV spreads rapidly, necessitating the development of sensitive diagnostic methods using biosensing devices. Biosensing strategies for dengue virus detection can be broadly categorized into optical, electrochemical, microwave, microfluidic, and CRISPR–based approaches. Optical biosensors include colorimetric sensors, surface plasmon resonance, surface–enhanced Raman scattering, and photonic crystal platforms. Electrochemical biosensors commonly employ techniques such as cyclic voltammetry, amperometry, and electrochemical impedance spectroscopy, while microwave biosensors primarily rely on split–ring resonator platforms. Microfluidic platforms integrate lab–on–chip architectures, and CRISPR–based assays exploit programmable nucleic acid recognition for molecular detection. Recently, artificial intelligence has been integrated with diagnostic technologies to enhance signal interpretation and clinical decision–making. Table 3 qualitatively compares the biosensor classes and their contributions to dengue diagnosis.

#### 3.4.1. Optical Biosensors

An optical biosensor is a compact analytical tool that contains a biological sensing unit coupled with an optical transducer. These biosensors provide a signal proportional to the analyte concentration [54]. Optical biosensors primarily detect photons instead of electrons. The variables measured using optical biosensors include reflectance, absorbance, and fluorescence. Several optical biosensors have been developed to detect DENV.

##### Colorimetric Biosensors

**Figure 6:**
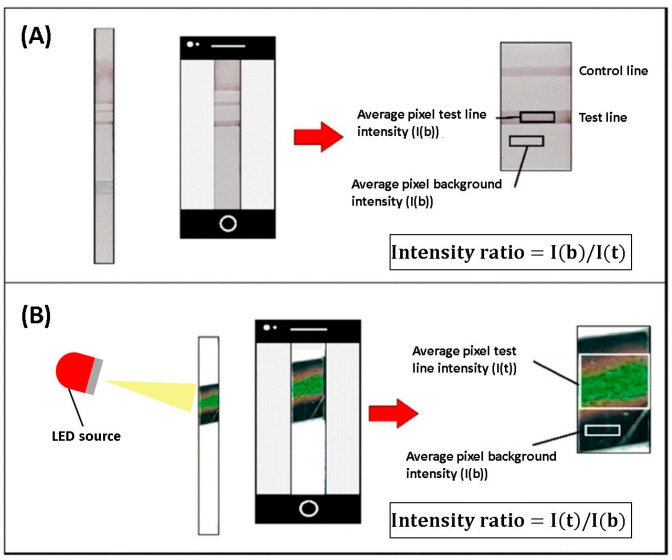
Schematic showing the differences between (**A**) a typical visual assay and (**B**) the proposed colorimetric thermal biosensor. Colors are used to illustrate illumination, thermochromic color change, and regions selected for intensity analysis. Adapted with permission from Ref. [55].

Colorimetric sensors are optical biosensors that produce a visible color change upon target binding. The sensing surface is functionalized with enzymes, nanoparticles, or dyes, and analyte binding alters optical properties. In many assays, the intensity of the color correlates with analyte concentration. Their acceptable sensitivity, selectivity, and ease of fabrication render them suitable for detecting biomolecules, gases, DNA, proteins, and viruses. DENV NS1 antigen was successfully detected using a colorimetric thermal biosensor. The biosensor utilizes an LFA and recombinant DENV–2 NS1 protein spiked into buffer and serum samples as a model antigen. Gold nanospheres (AuNSPs) were used as signal providers for LFA and as light–to–heat converters for detection by a thermal sensor. The assay was covered with a liquid crystal thermochromic sheet, which acted as a thermal sensor that displayed the color change, as shown in Figure 6. This method reduced the LOD by four times compared to typical LFA readouts, achieving an LOD of 1.56 ng/mL [55]. Increasing the LOD and visibility of colorimetric sensors to a level high enough to be detected by the naked human eye is one of the main challenges in developing these biosensors.

The thermochromic LFA represents a colorimetric signal amplification method that enhances sensitivity while maintaining the core advantages of conventional colorimetric LFAs, including simplicity, visual readout, and suitability for point–of–care applications. Although thermochromic LFA is presented here as a representative example due to these features, next–generation LFAs are increasingly dominated by quantitative approaches such as fluorescence–, magnetic–, and SERS–based platforms. While these methods offer improved sensitivity and quantitative capability, their reliance on external instrumentation may limit portability and point–of–care deployment. The thermochromic approach is therefore discussed as a complementary strategy that improves sensitivity with only a modest increase in system complexity, as the added components remain low–cost and portable. In applications requiring enhanced sensitivity, this trade–off can be justified, positioning thermochromic LFAs as a practical balance between sensitivity enhancement and operational simplicity.

##### Plasmonic Biosensors

**Figure 7:**
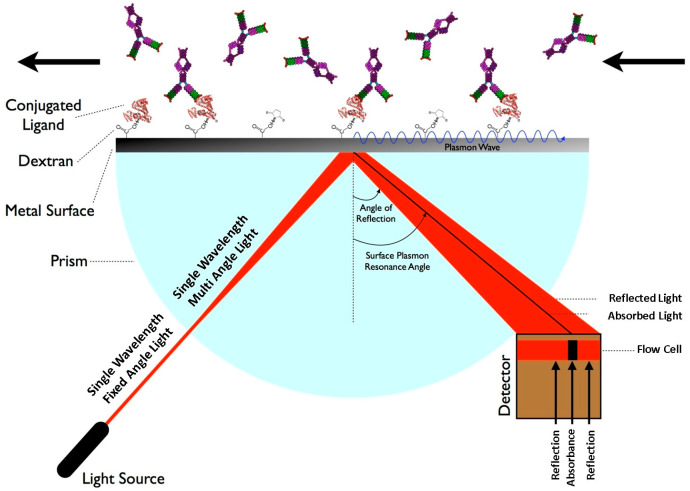
Schematic of the Surface Plasmon Resonance sensing principle. The arrows indicate the direction of light propagation, reflection, and analyte transport along the sensor surface. Reprinted with permission from Ref. [57].

Surface Plasmon Resonance (SPR) biosensors are optical biosensors that detect changes in surface plasmon waves when a biomolecule interacts with a biorecognition element. The biorecognition element interacts with the target analyte and produces a change in the refractive index at the surface of the sensor [56]. The variation in the propagation constant of the surface plasmon wave was measured to generate the readings. Research involving SPR biosensors has mostly relied on the Kretschmann configuration. The sensor film was the target of a p–polarized light beam in the Kretschmann configuration. Surface plasmons resonate when the tangential x–component of the incident light optical wave vector matches the surface plasmon optical wave vector, resulting in a dip in the reflected light (black line) at the incident angle. A Kretschmann configuration showing a basic SPR schematic is shown in Figure 7. The wavelength of the incident light beam is a concern in the field of SPR sensors.

**Figure 8:**
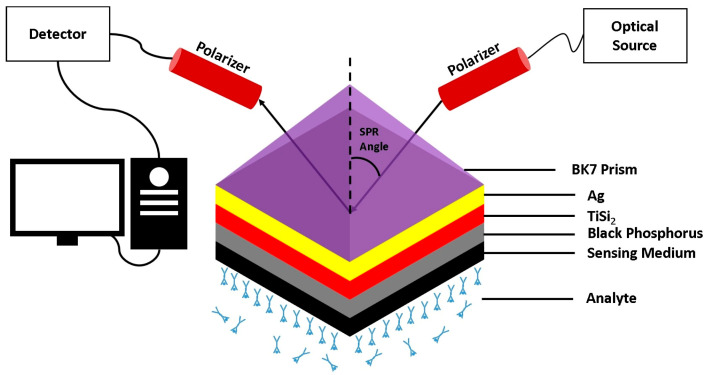
Schematic illustration of the proposed multilayer SPR sensor configuration based on the Kretschmann geometry. The structure consists of a BK7 prism, an Ag layer, a titanium disilicide (TiSi_2_) layer, a black phosphorus layer, and the sensing medium in contact with the analyte. Adapted with permission from Ref. [59]. Copyright 2023 Springer Nature.

In [58], a portable SPR–based platform was designed to diagnose DENV by detecting NS1–antigen. The platform uses a silver–coated optical fiber with a self–assembled monolayer of alkanethiols for antigen binding. Real blood serum was successfully tested, and the effectiveness of the platform was confirmed. An SPR–based biosensor made by layering the BK7 prism, Ag, titanium disilicide, black phosphorus, and sensing medium was proposed in [59]. The sensing medium comprised healthy and infected blood plasma, platelets, and hemoglobin. The sensor demonstrated a maximum reported sensitivity of 257.3 deg/RIU and a quality Factor of 85.45 deg^−1^, deeming it suitable for DENV antigen detection. A visual representation of the proposed sensor is presented in Figure 8.

The study in [60] proposed a localized surface plasmon resonance (LSPR) sensor that can detect different dengue serotypes and differentiate them from the zika virus using gold nanorods functionalized with DENV proteins. The biosensor can detect anti–DENV monoclonal antibodies in diluted DENV serum samples at an LOD of 0.001 ng/mL. In [61], DENV NS1 was demonstrated using gold/EDC–NHS/IgG SPR with NS1 monoclonal antibodies on the SPR component layer. The presence of the target antigen produced a measurable shift in the SPR angle due to changes in the local refractive index. The sensor performance was evaluated using simulated sensing media representing dengue–infected samples. SPR and LSPR both detect refractive index changes at metal interfaces; however, their system configurations differ significantly. Conventional SPR typically requires prism–based coupling and bulky optical components, limiting portability. In contrast, LSPR relies on localized plasmon resonances in metallic nanostructures, enabling compact, chip–scale sensors that are easily integrated into portable and point–of–care diagnostic platforms.

##### Raman–Based Biosensors

Surface–enhanced Raman scattering (SERS) is a detection technique in which the Raman scattering of different analytes can be enhanced by modifying the surface of the biorecognition element using various techniques or nanomaterials. A Raman spectrometer is shone on the biosensor surface, and the Raman scattering is measured. A laser beam, commonly at 532 nm, 633 nm, or 785 nm, illuminates the biosensor surface, and the inelastically scattered light is collected to measure molecular vibrations. Each analyte produces a characteristic Raman spectrum, which allows specific identification based on its scattering signature. SERS–based techniques have recently been implemented to differentiate between infected and healthy blood sera. A silver nanorod array (AgNRs) fabricated by glancing angle deposition was used as a SERS substrate to detect the presence of dengue with a mix of tested samples containing NS1– and IgM–positive using 5 µL of blood serum samples. Principal component analysis was used to verify the results and identify dengue–positive,–negative, and healthy individuals with great variability and maximum contribution. The proposed platform achieved an Enhancement Factor of 1.7 × 10^7^ and a reproducibility of 7.05% [62]. A SERS–based multiplexed lateral flow immunoassay made with encoded gold nanostars conjugated to specific antibodies (NS1) for each of DENV and Zika virus was successful in detecting dengue and Zika viruses. The assay was evaluated using serum samples, and dengue virus antigens were detected with an LOD of 7.67 ng/mL. This approach demonstrates the potential of SERS–integrated LFAs for early viral disease diagnosis [63].

##### Photonic Biosensors

**Figure 9:**
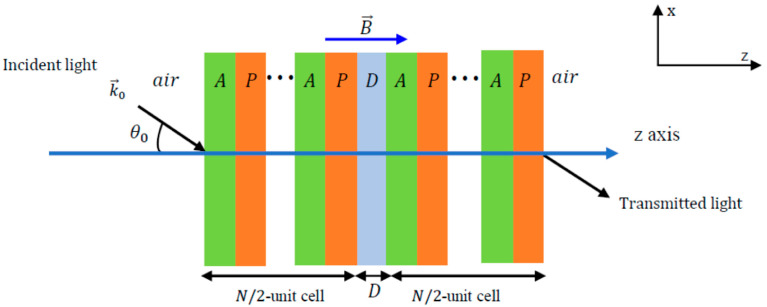
Schematic of a 1D plasmonic crystal configuration with defect layer. In blood sensing, the defect layer corresponds to the blood sample. The ellipses (“…”) indicate the periodic repetition of the A–P unit cells on either side of the central defect layer (D). Reprinted with permission from Ref. [65].

Photonic crystals are nanostructured materials arranged periodically, resulting in several different refractive indices. These structures have photonic gap bands that prevent photons from being refracted in certain directions, making them suitable for various applications. In biosensing, the photonic crystal surface is functionalized with specific bioreceptors, such as antibodies or aptamers. Binding of the target analyte changes the local refractive index, causing a measurable shift in the photonic bandgap. This wavelength shift serves as the detection signal, enabling label–free, sensitive, and quantitative detection of biomolecules [64]. Photonic crystals can have different spatial arrangements, resulting in 1D, 2D, or 3D arrangements, each of which can be used to determine different molecules. A 1D photonic crystal arrangement with a defect layer is illustrated in Figure 9. A defect layer was introduced to change the refractive index of the structure. This change can be detected and used in sensing applications.

The study in [66] proposed a 1D photonic crystal biosensor that can detect DENV using (Si/LiF)_6_D(LiF/Si)_6_ with D being the defect layer acting as an optical filter. The biosensor detects DENV based on the numerical shift in the transmission peak in the refractive index of infected blood. Challenges in the development of photonic crystals lie in the complexity of the design, which is not fully compatible with optical platforms requiring accurate and careful synthesis, and an incomplete photonic gap band [67].

#### 3.4.2. Electrochemical Biosensors

**Figure 10:**
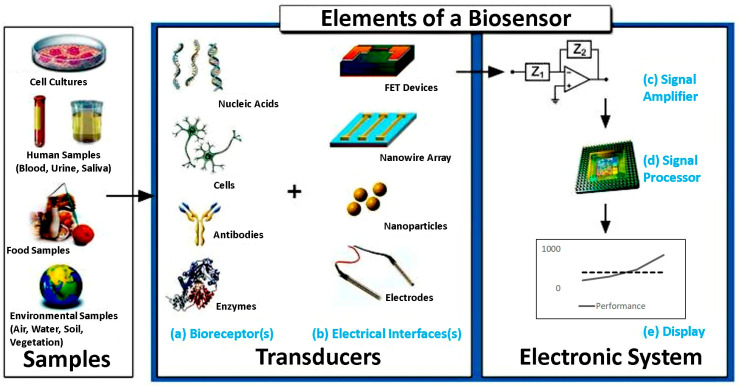
Common elements of a typical biosensor, including the sample (analyte). (**a**) The biorecognition components such as nucleic acids, cells, antibodies, or cells. (**b**) The electronics involved in processing and displaying the readout. (**c**) The signal amplifying element responsible for enhancing the generated signal. (**d**) The signal processor used for data analysis. (**e**) The display unit to visualize the output. Reprinted with permission from Ref. [68].

In electrochemical sensors, the molecular interactions between the biorecognition element and target analyte generate a change in the electrical signal (current, voltage, capacitance, or impedance) on the surface of the biosensor, which is measured and used to determine and quantify the biomolecule. Electrochemical biosensors can identify viral targets, making them suitable for DENV detection. Electrochemical biosensors have several components, such as a bioreceptor element specific to binding with the analyte, a platform on which the reaction takes place, a transducer element that converts the result of the reaction to electrical signals, and an electronic circuit that amplifies the signal to be converted into a format where it can be processed and then viewed in a readable format, as presented in Figure 10 [68]. The preferred design for electrochemical sensors consists of three electrodes: an anode, cathode, and reference electrode. The most commonly used electrochemical sensors are based on cyclic voltammetry, chronoamperometry, and impedance spectroscopy.

**Figure 11:**
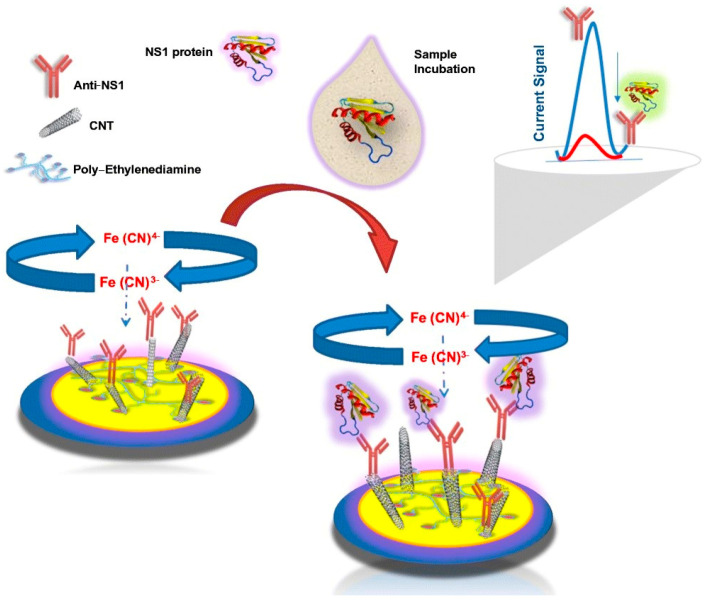
Schematic illustration of the proposed electrochemical immunosensor for NS1 antigen detection showing surface functionalization and the signal transduction mechanism. The current signal plot illustrates the change in electrochemical response before and after NS1 antigen binding. Reprinted with permission from Ref. [69]. Copyright 2021 Springer Nature.

Cyclic voltammetry measures the current flowing between two electrodes, one working and one counter, that are exposed to a voltage differential during a redox chemical reaction. The redox current is controlled by the working electrode, whereas the counter electrode maintains a constant voltage while passing a current to counteract the redox events at the working electrode. Making this setup work is challenging; thus, a third electrode, known as the reference electrode, is added to relieve the counter electrode from maintaining the voltage differential. Differential pulse voltammetry (DPV) is another voltammetry variation that determines the current variation as a function of voltage. Voltammetry can be used to detect viral molecules, such as DENV. An immunosensor designed to detect DENV NS1 antigens in human serum and urine samples and ZIKV using carbon nanotube–ethylenediamine was reported to have an LOD of 6.8 ng/mL. The biosensor uses an assembled thin film of carbon nanotube–ethylenediamine with anti–NS1 monoclonal antibodies immobilized on the carbon nanotube, as demonstrated in Figure 11, and DPV was used to determine the analytical response to NS1 [69].

A biosensor using two sensing techniques, composite AuNPs and nitrogen and sulfur co–doped graphene quantum dots (N,S–GQDs), was developed to identify different DENV serotypes. DNA was quantified using DPV, and the serotypes were successfully identified using synthetic DNA targets in buffer solutions with great efficiency and a detection limit in the femtomolar range [70].

Amperometry and chronoamperometry, similar to voltammetry, use the current measured at the working electrode concerning the reference electrode. It uses a fixed potential applied to the working electrode and measures the oxide reduction reaction current. Amperometry differs from voltammetry in that its temporal resolution is not limited to the duration of the cycle. Therefore, it can be used to measure the kinetics of the viral molecules. Chronoamperometry was used to assess the effectiveness of graphene oxide (GO) nanostructures functionalized using a ruthenium bipyridine complex (Ru(II)). Monoclonal antibodies (mAbs) were immobilized using a bioaffinity layer (Pro–GO). The complex (GO–Ru(II)/Pro–G/mAb) was shown to have an LOD of 0.38 ng/mL and sensitivity of 0.14 µA/ng mL^−1^ evaluated using serum samples from the detection of DENV NS1 [71]. The study in [72] described a needle–like Cu_2_CdSnS_4_ quaternary alloy nanostructure deposited on an oxygen–etched silicon substrate (O^2^/Si) based biosensor for the amperometric detection of DENV DNA. The biosensor was evaluated using synthetic DNA targets in buffered solutions and achieved an LOD of 17 nM and a calculated sensitivity of 24.2 µA/nm cm^−2^. The quaternary alloy helps in the immobilization of DENV–specific DNA, and the electrodes are made by depositing silver on the quaternary alloy substrate.

**Figure 12:**
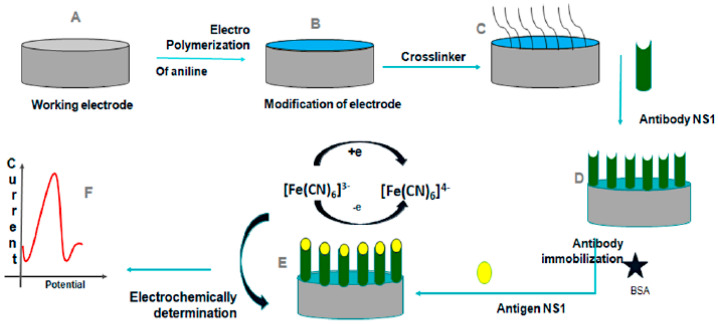
Schematic illustration of the electrochemical detection of DENV using a polyaniline (PANI)–modified glassy carbon electrode. (**A**–**C**) Electrode preparation and surface modification, including aniline polymerization and crosslinker attachment. (**D**) Immobilization of anti–NS1 antibodies and surface blocking with bovine serum albumin (BSA). (**E**,**F**) NS1 antigen binding and electrochemical signal generation through changes in the redox probe response measured by EIS. Different colors distinguish the electrode surface, polymer layer, immobilized antibodies, and redox probe. Reprinted with permission from Ref. [75].

Electrochemical impedance spectroscopy (EIS) functions as a transfer function by measuring the impedance and capacitive properties of the substrate based on the applied operating frequency. EIS can provide information on the electrical, electrochemical, and physical properties of analytes. When a potential is applied, the electrode exhibits a baseline impedance. Binding of the target analyte forms a non–conductive layer on the electrode surface, increasing charge–transfer resistance and altering capacitance. These measurable changes serve as the detection signal. Its low cost play a major role in its widespread use as a detection method in semiconductor science, biosensing, and noninvasive diagnostics. Blood samples containing viral molecules have different impedances and capacitances, which can be identified by EIS [73]. Developments in EIS biosensors involve the integration of new designs and materials into the working electrode. A multiplex biosensor using EIS was designed to detect Dengue and Zika infections using a single chip with NS1 target molecules for both viruses. The working electrode was made using the self–assembled monolayer technique, and the gold surface was modified with cysteamine and DENV NS1 monoclonal antibodies for the dengue detection electrode. Electrochemical detection was validated using serum samples on a single chip and achieved an LOD of 1.17 ng/mL, providing a potential portable biosensor for POC diagnosis [74]. Glassy carbon electrodes coated with modified polyaniline (PANI) with NS1 antibodies detected dengue fever with an LOD of 0.33 ng/mL and was evaluated using NS1 antigen spiked into buffer and serum samples. The designed sensor had great reproducibility and was economical, making it suitable for POC detection [75]. A visual representation of the approach is shown in Figure 12.

#### 3.4.3. Microwave Based Sensors

**Figure 13:**
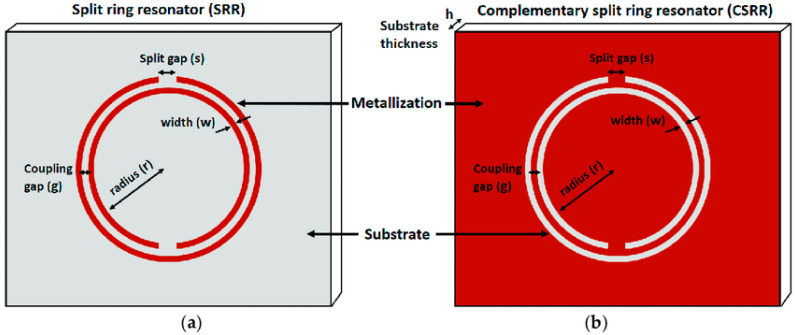
Basic configuration of (**a**) a double–ringed split–ring resonator and (**b**) its complementary structure. Reprinted with permission from Ref. [77].

Microwave sensors are used in many disciplines, such as biomedical sensing, meteorology and weather monitoring, and the chemical industry. The discovery and introduction of metamaterials have played an important role in the development of the microwave field by improving the sensitivity of sensors and decreasing their size. Owing to their ability to interact with cells noninvasively, microwave biosensors may provide promising outcomes in biomedical applications. Microwave biosensors are designed using transmission lines loaded with planar resonators. The resonator acts as a band–stop notch in the transmission and reflection responses. The sensed stimuli (in this case, blood) can modify the resonance properties, such as the notch frequency, depth, and/or quality factor. The detection of electromagnetic (EM) waves in the frequency range of gigahertz (GHz) and even up to terahertz (THz) has been described as a successful method for characterizing biological suspensions and biomaterials. The benefits of microwave biosensors are their low cost, small size, and light weight, which have led to their widespread application in many biomedical sensors. Most of these methods employ a microstrip line to measure variations in the dielectric substrate to enhance treatment plans and reduce fatal complications. Developments in the field of split–ring resonators (SRRs) have yielded favorable results for DENV detection. SRRs and their complementary configurations (CSRRs) are made using metamaterials and can exhibit negative permeability and negative refractive indices when a perpendicular magnetic field is incident on the SRR plane [76]. A common design among split–ring resonators is a double–ringed structure with a gap in each ring in opposite directions. Figure 13 shows the basic topologies of the SRRs and CSRRs.

By linking four identical SRRs to a graphene ring, a microwave–based SRR biosensor was proposed. The sensor exhibited absorption at 1.354 THz, and its sensitivity could be tuned by adjusting the chemical potential of the graphene ring, a sensitivity of 1.7 THz/RIU and a Figure of Merit of165.09 RIU^−1^ were attained. The device performance was evaluated using simulated dielectric environments representing analyte–induced refractive index changes, demonstrating potential applicability for detecting viral molecules, including dengue [78]. DENV infection can be characterized by a decreased number of platelets in the blood. Dengue infection is commonly associated with thrombocytopenia and coagulation abnormalities caused by immune–mediated platelet destruction, bone marrow suppression, and vascular leakage [79,80,81]. These pathological changes alter blood composition, including platelet concentration, plasma protein content, and hematocrit [82,83]. A reduced platelet count and increased coagulation lead to changes in ionic mobility and plasma conductivity, resulting in a decrease in blood permittivity. As coagulation progresses, hematocrit levels increase, further modifying the dielectric properties of blood. Blood permittivity exhibits the same properties as capacitance, which can be measured using SRRs. A microwave SRR biosensor was proposed in [84] and contained two parts: a removable/disposable resonator and a fixed transmission line. The resonator was made removable so that it could be replaced or cleaned, allowing it to be reused. The resonator and transmission line were magnetically excited to form a mutual conductance and capacitance. The sensor was experimentally validated using blood samples with controlled dielectric properties corresponding to varying platelet concentrations, establishing a strong correlation between resonance shifts and blood permittivity. The presence of mutual conductance and capacitance increases the overall conductance and capacitance of the system and exhibited a sensitivity of 0.325 GHz with an R^2^ value of 0.9729.

#### 3.4.4. Other Biosensors

This section covers biosensors utilizing microfluidic chips and Clustered regularly interspaced short palindromic repeats (CRISPR)–based genome editing technology.

##### Microfluidic Biosensors

**Figure 14:**
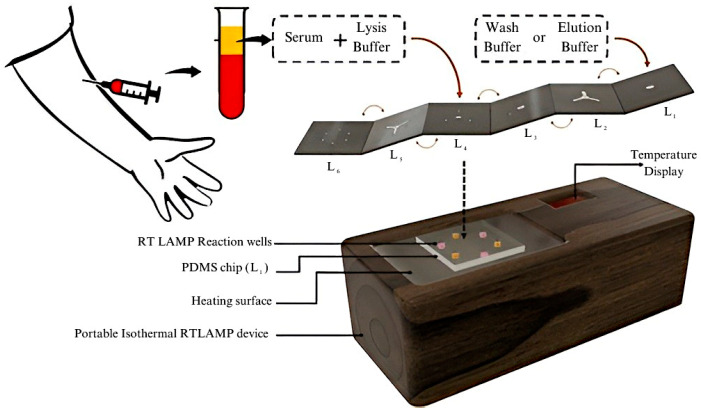
Schematic of proposed 7–layer microfluidic sensor based on reverse transcriptase—loop mediated isothermal amplification (RT–LAMP). Reprinted with permission from Ref. [85]. Copyright 2022 American Chemical Society.

Microfluidic chips contain a set of microchannels engraved on different materials, and different microchannel arrangements are designed to achieve the desired results. The flow of fluid in these arrangements provides information about the analytes present in the sample. Microfluidic chips can be arranged in 2D, 3D, or 4D configurations, and their integration with biorecognition elements or antibodies is the basis of microfluidic sensors. The high surface area, sensitivity, cost–effectiveness, portability, and rapid detection time of these biosensors make them of emerging interest among researchers. Microfluidic chips can be integrated with different detection techniques to create a lab–on–a–chip (LoC) complex, which greatly improves the ability to detect the target analyte. LoC devices integrate several steps into a single platform, called a chip. Biochemical reactions, transportation, sample concentration, and detection can all occur within these devices. This makes LoCs robust, portable, and rapid in detecting target molecules. Since many techniques can be implemented, LoCs can detect a wide variety of molecules, such as IgG/IgM antibodies, NS1 antigens, RNA, and even E proteins. These characteristics make the devices suitable for dengue fever detection. Biosensors with DENV–specific immobilized antibodies can be used to detect dengue infection. An all–in–one, 7–layer, portable microfluidic biosensor made using multilayered Origami–based Paper/Polymer synchronizing nucleic acid isolation, isothermal amplification, and colorimetric analytics was designed to detect all four serotypes of DENV. The 6 layers are made to separate serum from the blood, and the 7th layer uses the reverse transcriptase–loop mediated isothermal amplification (RT–LAMP) technique to identify the presence of dengue serotypes. The detection of dengue is performed in 5 min, this fast, quick and portable method can be implemented in POC testing in the future [85]. The proposed system is visualized in Figure 14. LAMP differs from conventional PCR in that it is more specific and takes less time to provide results. A microfluidic biosensor made of Zinc Oxide nanorods (ZnO–NR) using seed–assisted hydrothermal synthesis can detect DENV in serum samples with an LOD of 3.1 × 10^−4^ ng/mL for DENV–3. ZnO–NRs have high purity and surface–to–volume ratio, which creates an abundance in binding sites and has an improved functionalization efficiency with 4G2 monoclonal antibodies [86].

##### CRISPR Based Assays

**Figure 15:**
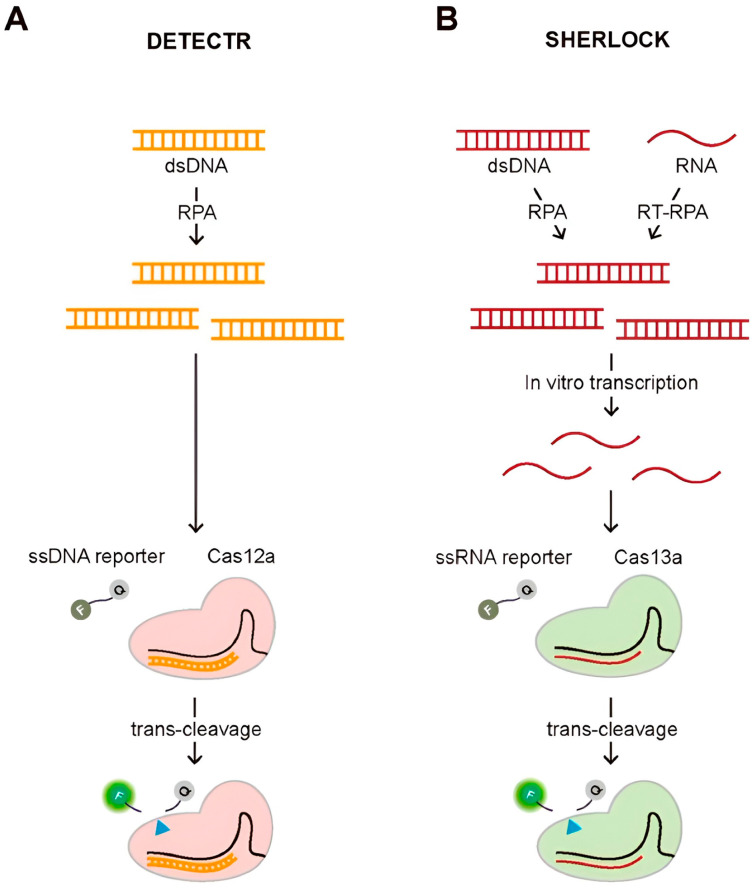
(**A**) Schematic of DETECTR and (**B**) Schematic of SHERLOCK diagnostic methods. The blue triangle indicates the cleavage site on the reporter molecule during Cas–mediated trans–cleavage. Reproduced with permission from Ref. [87].

CRISPR–based assays, including Specific High–sensitivity Enzymatic Reporter un–locking (SHERLOCK) and DNA endonuclease–targeted CRISPR Trans reporter (DETECTR), use RNA–guided Cas proteins to specifically recognize target nucleic acids. Upon binding, the activated Cas enzyme cleaves a reporter molecule, generating a fluorescent or colorimetric signal. The signal intensity correlates with target concentration, enabling rapid, sensitive, and specific detection of RNA or DNA in clinical samples [87]. An illustration of these diagnostic methods employing trans–cleavage activity is shown in Figure 15.

An electrochemical biosensor using a CRISPR system and methylene blue–conjugated Au nanoparticles was designed for DENV detection, demonstrating enhanced sensitivity. The sensor uses a CRISPR/Cpf1 system to recognize target sequences and measure dengue viral RNA. The biosensor demonstrated ultrasensitive detection of DENV–4 at concentrations as low as 100 fM using synthetic viral RNA targets in buffered solutions, without the need for nucleic acid amplification methods. The CRISPR/Cpf1 system is a class 2 CRISPR system that consists of a Cas protein conjugated to crRNA. It recognizes and cleaves target DNA without a protospacer proximal motif (PAM) sequence, unlike Cas9 and Cas13a. Electrophoresis has been used to analyze the presence of dengue [88]. By combining CRISPR with other nucleic amplification techniques, such as RT–PCR, cross–contamination can be reduced. A proximity sequence–enhanced CRISPR–Cas12a biosensor was devised and demonstrated its use using spiked buffer samples for the detection of DENV. The biosensor is connected through a Hybridization Chain Reaction (HCR), an isothermal nucleic acid amplification technique. The ssDNA was labeled with a fluorophore and quencher at each end (ssDNA–FQ) and generated fluorescence signals upon target recognition. The proposed design achieved an LOD of 51fM, making it suitable for clinical applications [89]. CRISPR–Cas13a has also been used to successfully identify dengue infection, and by combining this technique with amperometry, an LOD of 0.78 fM was achieved and validated using synthetic RNA targets [90].

The difference in CRISPR techniques is their cutting manner and targets; Cas9 creates breaks in the ssDNA, creating variable insertions and deletions. While Cas12 cuts DNA in a staggered manner, creating more consistent repairs. Similarly to Cas9, Cas13 is guided by an RNA nuclease; however, it exclusively targets single–stranded RNA, as opposed to ssDNA. A general comparison of the sensing technologies used, their contributions, and their disadvantages is presented in Table 4.

As biosensor systems generate increasingly complex electrical and optical signals, artificial intelligence has been incorporated to improve signal interpretation, noise reduction, and diagnostic accuracy.

##### Artificial Intelligence

**Figure 16:**
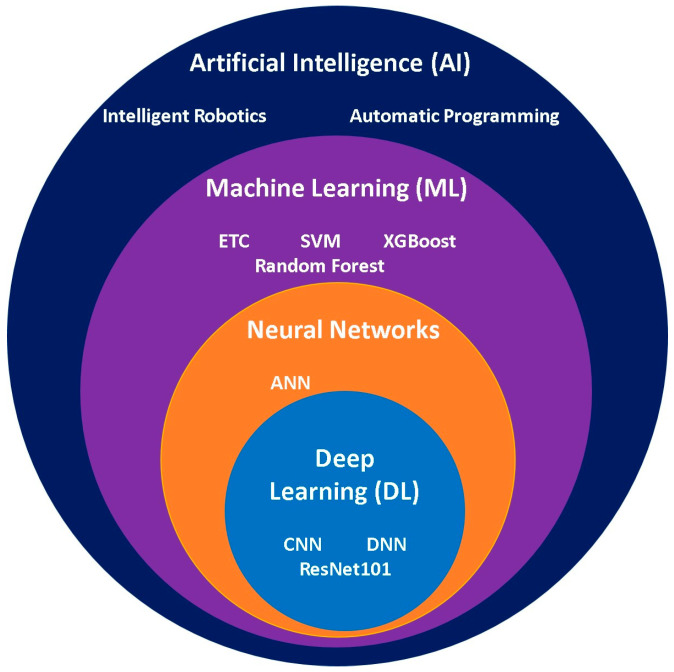
Euler diagram illustrating the hierarchy of artificial intelligence learning algorithms and their distinct characteristics.

The rapid development of artificial intelligence (AI) has enabled its application in disease diagnosis and the assessment of disease progression using previously collected patient data. Relevant features are extracted from clinical datasets and classified according to the suspected disease or infection, after which machine learning (ML) algorithms are trained to create predictive models that can identify diseases. The datasets used for training ML models are typically pre–processed and cleaned to improve model performance and prediction accuracy. The emergence of deep learning (DL) has created promising opportunities in the field of AI by enabling automated feature extraction and improved pattern recognition. The Hierarchical relationship between AI, ML, and DL algorithms is illustrated in Figure 16.

Several ML and DL models have been used to detect dengue. Many factors affect the accuracy of prediction, such as the ML or DL model used, data preprocessing, sample size, sample collection period, selected features, statistical tests performed, and hyperparameter tuning. A study conducted to predict the early detection of DENV using complete blood count (CBC) data implemented a stack ensemble classifier based on several ML models, which provided high detection accuracy. A total of 320 samples comprising 14 hematological features were used as a dataset for several ML and DL models, of which the data were divided into an 80% training set and 20% testing set (80%:20%). Five statistical tests were used among the different ML and DL models, and hyperparameter optimization was performed using GridSearchCV for ML and Keras Tuner frameworks for the DL models. The accuracies of each ML and DL model were compared, and the proposed stack ensemble classifier made using multilayer perceptron artificial neural network (DL), Extreme Gradient Boosting (ML), and Logistic Regression (ML) with light gradient boosting machine (ML) as a meta–classifier had the highest accuracy of 96.88%, outperforming the other tested models and providing a means for the early detection of dengue fever [103]. It was found in [104] that Support Vector Machine (SVM) performed better than other ML models using routine clinical data, a database comprising 300 suspected cases of which 184 patients were PCR positive for DENV–1,2,3. Hematological and Serological features (presence of NS1, IgG, and IgM) and demographic information were used as inputs for the ML models. SVM exhibited an accuracy of 71.4% in predicting dengue PCR results, paving the way for the inclusion of ML to aid in dengue diagnosis [104]. Another study found that the Extra Tree Classifier (ETC) was the best performing, achieving an accuracy of 99.12%. The study used a database of 6694 samples with 21 features based on clinically reported symptoms such as headaches, fever, and general weakness [105]. The study in [106] used seven ML algorithms with fixed features of clinically reported symptoms combined with three blood count metrics (hemoglobin, white blood cells, and platelets) and reported that the LogitBoost ensemble model outperformed the others and had a classification accuracy of 92%. It was also noted that fever and hemoglobin significantly affected the decision–making process. A study was conducted on the use of AI in diagnosing dengue infection using a dataset of 21,157 cases collected from a public hospital in Peru between January and July 2023. Eight dataset features based on clinically reported symptoms were used for the Support Vector Machine (SVM), Artificial Neural Network (ANN), and random forest algorithms, and their performances were compared. It was reported that SVM had the highest sensitivity of 99.05%, whereas ANN had the best overall performance with an accuracy of 86.47% and a recall of 92.91% [107]. In [108], a study conducted in China comprising a database of 4894 cases using only four features made of a mix of clinically reported symptoms and CBC characteristics concluded that the deep neural network (DNN) had the best overall performance with an area under curve (AUC) of 85.87 ± 0.54% and the logistic regression (LR) model showed that it had the high sensitivity of 93.1%. The use of only four features signified the strength of using neural networks for dengue prediction.

Other approaches to DENV prediction have also been implemented, including the implementation of a model that aids in predicting blood component transfusion for hospitalized patients. Eight predictive models were developed, and 16 significant features based on CBC were used. The database was collected from 1148 patients daily until their discharge or death. XGBoost exhibited the best performance, with an AUC of 79.3% [109]. An approach to identify DENV–infected patients from healthy ones using spectroscopic images was performed by exploiting the classification criteria of the ResNet101 DL model by applying the concept of transfer learning, yielding an accuracy of 96%. The dataset included 2000 Raman spectra images of 100 suspected patients (20 images per patient) [110]. A random forest algorithm was implemented to assess misdiagnosed dengue hospitalization and to predict the regional spread of the virus. The database consists of meteorological data and dengue incidence in a binary (yes/no) manner and comprises 400,202 entries collected from 2014 to 2020. The random Forest achieved an accuracy of 85% [111]. Table 5 summarizes representative state–of–the–art AI models used for dengue infection prediction and their reported classification metrics.

Handling missing data input is crucial for preprocessing. In [103], median imputation was used to address the missing features, which could have been done to preserve the dataset size but could add unwanted uncertainty instead of discarding them [112]. The mean method was implemented to address missing values [105]. The study in [100] used ReplaceMissingValues, a Waikato Environment for Knowledge Analysis (WEKA) tool that replaces missing values with the modes and means from the training data [113], which could weaken the feature correlations due to bias. Entries with missing data were excluded in [104,108]. In [109], the missing data were handled using regression imputation, while fields with more than 50% missing data were excluded. Since [110] uses a fundamentally different approach, data handling follows a different procedure that was not stated in the paper. The data handling techniques were not stated in [111].

Because of the different stages of dengue infection (suspected, febrile, critical, and recovery), it can be challenging to apply a standardized ML/DL model to accurately detect the onset of the disease as model performance is strongly influenced by the disease stage at which the data is collected. Therefore, it is important to state the disease stage at which the ML models were implemented to properly assess and compare their performances.

Beyond clinical prediction, AI can be applied directly to biosensor signal processing to improve analytical performance. In electrochemical impedance spectroscopy, machine learning can enhance signal–to–noise ratios through denoising and feature extraction, which lowers the limit of detection. Similarly, in optical techniques such as SERS and SPR, AI–based spectral analysis enables automated feature extraction and classification of complex spectral signatures, as demonstrated in [110]. For microwave sensors, pattern recognition models can be used to distinguish subtle resonance shifts caused by the dielectric changes in analyte concentrations. Integrating AI with biosensor signal processing enhances sensitivity, selectivity, and robustness in next–generation diagnostic platforms.

Researchers have developed different ML and DL models to aid physicians in accurately diagnosing dengue fever. In addition, differences in feature selection, hyperparameter tuning, preprocessing methods, and missing data handling contribute to performance variability across studies. The use of inconsistent validation strategies and performance metrics may lead to model overfitting and inflated accuracy values, limiting its general application. Despite these variations, it was noted that hemoglobin, platelet count, white blood cell count, and the presence of fever were consistently decisive in differentiating positive from negative cases, proving their importance in accurate clinical diagnosis.

## 4. Future Outlooks and Conclusions

The diagnostic technologies reviewed illustrate a shift from centralized laboratory testing toward portable, data–driven, and point–of–care dengue detection systems. It is estimated that 400 million people are at risk of DENV infection per year, with testing and diagnosis being the main methods to alert people about the emergence of outbreaks in affected areas. The rise in social media use in recent years has significantly impacted our daily lives. In areas with inadequate medical resources, social media–based platforms can facilitate health communication and alert people to outbreaks. However, the spread of misinformation has also become common because regulation is very difficult; therefore, individual alertness is required while browsing media outlets. The initial diagnosis of dengue fever is usually performed by assessing the reported symptoms. Since this is not enough to determine the type of fever a patient has, advances in diagnostic technologies are of utmost importance. New technologies and sensing techniques can decrease the possibility of misdiagnosis and help in early diagnosis. Most biosensing techniques are based on the first onset of symptoms, that is, after 5 days of contracting DENV. Misdiagnosis of suspected cases is a serious issue that needs to be mitigated, which calls for immediate confirmatory diagnosis of the disease to avoid the progression and onset of severe symptoms and, in the worst–case scenario, the development of dengue hemorrhagic fever. Most diagnostic procedures are either performed in laboratory or clinical settings, requiring expensive equipment, are time–consuming, and require skilled laboratory technicians. The long time required to prepare samples, long culturing periods, cross–contamination, and the inability to differentiate between serotypes are among the most challenging aspects of properly diagnosing DENV. The development of biosensors has introduced modern technologies that are fast, inexpensive, robust, portable, sensitive, and disposable and can be applied to POC testing. Additionally, recent biosensing studies have demonstrated real–time detection platforms capable of continuous and rapid signal acquisition, further supporting the need for timely diagnostic tools in early disease management [114]. Furthermore, the implementation of AI algorithms has helped verify and predict the results obtained by these methods. This article presents some advancements in biosensor development and detection strategies, listed in order of publication year in Table 6.

## Figures and Tables

**Table 1:** Dengue analytes and their descriptions.

Analyte	Detection Biofluids	Description	Concentration Range *
NS1 Antigen	Serum, plasma, whole blood	Viral Non-structural protein released during early infection	~1–50 ng/mL
DENV–2 NS1	Serum, plasma	Serotype—specific NS1 antigen	~1–30 ng/mL
Viral RNA	Serum, plasma, whole blood	DENV Genomic RNA	~10^2^–10^6^ copies/mL
Anti—DENV IgM	Serum, plasma	Host Antibody released following first infection	Detected after the 4th or 5th day after the onset of infection symptoms
Anti—DENV IgG	Serum, plasma	Host Anitbody release following second infection	Elevated after secondary infections
DENV RNA in Urine	Urine	Viral RNA detected at later stages of infection	~101–104 copies/mL

* Reported concentrations vary with infection stage and patient condition.

**Table 2:** Comparative table of standard clinical approaches to confirm dengue infection.

Method of Detection	Advantages	Disadvantages	Example(s)
Cell Culture Isolation	Reliable and accurate. Different serotypes can be identified by combining them with molecular or immunological assays [36].	It takes a long time to obtain results. Skilled personnel are required to perform the procedure. It should be coupled with other equipment to obtain meaningful readings. Cannot tell if the patient was previously infected with DENV or not [35,36].	C6/36 cell culture [22].
Genomic Detection (PCR)	Results can be obtained in less than 24 h [37]. It has a very high specificity and sensitivity [38,39]. It can be observed in real–time [40].	Cross–contamination can result in false–positives. It may not be available in poor areas because it requires expensive equipment to perform tests [41]. Sensitivity may decrease over time [28].	RT–PCR, RT–qPCR, and plasmonic PCR [30].
Serological Testing	Easy to perform. Can tell if a patient has been infected before [42]. It is very accurate in confirming infection. Results are rapidly achieved [43].	The type of flavivirus infection cannot be identified because of the same antibodies released. Low IgM levels may not properly indicate whether a patient was infected before and by which flavivirus [44].	LFA, immunofluorescence assays, and ELISA [23,31,32,33].

**Table 3:** The different biosensor classes and their advantages and limitations for application in this field are discussed.

Biosensor Types	Advantages	Limitations	Example(s)
Optical Biosensors	Specific, sensitive, rapid, and can be performed in real–time [46].	Not always portable, and may require complex equipment [47].	Colorimetry, surface plasmon resonance, surface enhanced Raman spectroscopy, photonic crystals.
Electrochemical Biosensors	Requires a small sample volume, is inexpensive, easy to use, and has high sensitivity.	Those sensors suffer from surface fouling, signal drift, and susceptibility to interference [48].	Cyclic Voltammetry, (DPV), chronoamperometry, amperometry, and EIS
Microwave–based Biosensors	High sensitivity, portable, lightweight, and has a wide range of operating frequencies.	Fabrication must be performed carefully and humidity, temperature, and the frequency range used can affect the results [49].	SRRs.
Microfluidics	Uses a smaller sample quantity, is inexpensive, and has the potential to be applied in point–of–care diagnosis [50].	Lab–on–chip modules can suffer from clogging due to their small tube and compartment sizes, and access to 3D printing and materials is needed [51].	LFA, lab–on–chip.
CRISPR Based Biosensors	Fast, state–of–the–art technology with ultra–high sensitivity [52].	Binding may occur at the wrong sites, leading to unwanted modifications [53].	CRISPR/Cpf1, CRISPR/Cas9, CRISPR/Cas12a, CRISPR/Cas13a.

**Table 4:** General Comparison between the advantages and disadvantages of the biosensing approaches discussed in this paper.

Method of Detection	Advantages	Disadvantages
Colorimetry	Simple to use, quick, and has acceptable specificity and sensitivity [91].	May require large concentrations, has a limited wavelength measurement range, and cannot analyze colorless substances [92].
SPR	Small sample size is required, real–time detection capability, and quick [93].	Portability of components, low sensitivity, sophisticated setup, and wavelength of incident light should be considered [94].
SERS	Provides highly specific signals with strong amplification and rapid detection capability	Substrate reuse is challenging, expensive, and poorly quantified [95].
Photonic Crystals	Efficient, can precisely control wavelength, and are compact, and sensitive [96].	Complex design, incomplete photonic bandgaps, and careful synthesis is needed to avoid incompatibility [97].
DPV	Rapid, inexpensive, and has superior sensitivity [98].	It is complex to set up and difficult to analyze the output.
Amperometry	High selectivity and sensitivity, is inexpensive, and has a low detection limit.	Long response time, sample preparation, and a continuous power supply is required [99].
EIS	Cost–effective, highly sensitive, and requires a low sample volume [100].	May damage samples, requires skilled personnel for preprocessing.
SRR	High sensitivity, portability, dielectric permittivity can be observed, and repeatability is possible.	Environmental changes may alter the output, a complex fabrication process is required, and losses may occur [76].
Microfluidics	Fewer samples are needed, and it has high sensitivity, low cost, and fewer reagents can be applied in POC [101].	Their fabrication may be expensive, suffer from clogging, and have limited sensitivity [49].
LFA	Easy, user–friendly, cheap, and fast [102].	The results are qualitative, with risk of cross–contamination and limited sensitivity to design.
CRISPR	Extremely sensitive, inexpensive, and rapid [52].	Unintended modifications and ethical concerns [53].

**Table 5:** Summary of state–of–the–art AI models Used to predict dengue infection.

Reference	Algorithm(s)	Dataset Size	Dataset Type	Description	Precision	Recall	Accuracy
[103]	Stacking Ensemble	320	CBC	Stacking ensemble classifier (LightGBM, XGBoost, Logistic Regression, and Multilayer perceptron learners).	0.9773	0.9545	0.9688
[104]	SVM	300	Demographic information, Serological tests, and CBC	Radial basis function kernel in Support Vector Machine (RBF—SVM).	0.7200	0.9700	0.7140
[105]	ETC	6694	Clinically reported symptoms	Five different efficient machine learning techniques were implemented and ETC was the highest–performing in all metrics.	0.9918	0.9912	0.9912
[106]	LogitBoost	75	Clinically reported symptoms and blood tests	An Ensemble classifier was applied to LogitBoost.	0.9500	0.9000	0.9200
[107]	SVM, ANN	21,157	Clinically reported symptoms	SVM had the highest sensitivity but ANN overall had better metrics based on AUC. The metrics listed are for the ANN model.	0.8647	0.9291	0.8647
[108]	DNN, LR	4894	Clinically reported symptoms and CBC	DNN had the best overall AUC, whereas LR showed the highest sensitivity.	N/A *	N/A	N/A
[109]	XGBoost	1148	CBC	Data was collected daily until discharge or death. XGBoost had the highest AUC. The study focused in the need for blood transfusion.	0.8210	0.6320	0.8720
[110]	DENV–TLDNN	2000	Raman spectra of blood sera	Transfer–Learning Resnet101.	0.9610	N/A	0.9600
[111]	Random Forest	400,202	Meteorological data and binary dengue infection (yes, no)	The study focused on the regional spread of dengue and its misdiagnosis in epidemic regions.	0.7306	0.9900	0.8500

* N/A: Not reported in the study.

**Table 6:** Summary of the detection methods discussed with their novel contributions and performance metrics organized by year of publication *.

Reference	Year of Publication	Method of Detection	Target Analyte	Sample Matrix	Limit of Detection (LOD)	Novel Work	Performance Metrics
[63]	2017	SERS	NS1	Serum	7.67 ng/mL	Multiplexed lateral flow assay with SERS–encoded gold nanostars.	High sensitivity was reported.
[72]	2017	Amperometry	ssDNA	Synthetic DENV–2 DNA in buffered solutions	17 nM	Cu_2_CdSnS_4_ quaternary alloy nanostructure biosensor deposited on O_2_/Si substrate.	Transduced current vs. DNA concentration: R^2^ = 0.5059 Calculated Sensitivity = 24.2 µA/nm cm^−2^ Standard deviation = 0.34 µA Limit of Quantification = 53.6 nM
[70]	2018	DPV	DNA	Synthetic DENV DNA in buffered solutions	9.4 fM	gold nanoparticle nanocomposite (AuNP) and nitrogen, sulfur co–doped graphene quantum dots (N,S–GQDs) with fluorescence technique.	Concentration range = 10^−14^–10^−6^ M Can identify different serotypes
[62]	2020	SERS	NS1	Blood serum	N/A	Surface–enhanced Raman spectroscopy was directly applied, and statistical analysis was performed using multivariate principal component analysis.	Variance and Cumulative percentages of the first and second principal components in PCA analysis. Enhancement Factor = 1.7 × 10^7^ Reproducibility = 7.05% Acquisition time = 30 s
[71]	2020	Chronoamperometry	NS1	NS1—Spiked serum samples	0.38 ng/mL	Opto–electrochemical functionalization of a ruthenium bipyridine complex on the surface of graphene oxide immunoprobe.	Amperometric current percentage vs. NS1 concentration: Correlation coefficient = 0.9976 Sensitivity = 0.14 µA/ng mL^−1^
[75]	2020	EIS	NS1	buffer and NS1—Spiked serum	0.33 ng/mL	A modified polyaniline–coated Glassy Carbon electrode was immobilized with DENV antibodies.	Concentration range = 1–100 ng/mL Calibration curve R^2^ = 0.997 Sensitivity (Slope) = 13.8% I_P_R/mL. ng^−1^ Stability < 5% Relative Standard Deviation = 1.9% (as a measure for reproducibility)
[60]	2020	LSPR	DENV E proteins	Diluted serum	0.001 ng/mL	Localized surface plasmon resonance coupled with gold nanorods functionalized with DENV E proteins.	The sensitivity and specificity to ELISA were calculated for each DENV serotype; sensitivities of 80.2%, 59.5%, 71.6%, and 69.1% were achieved for DENV 1, DENV 2, DENV 3, and DENV 4, respectively. Calculated specificities of 93.7%, 93.0%, 85.5%, and 91.5% were obtained for DENV 1–4 serotypes.
[69]	2021	DPV	NS1	Serum and urine	6.8 ng/mL	Nanostructured thin film carbon nanotube–ethylenediamine label–free immunosensor.	Linear range = 20–800 ng/mL R^2^ = 0.9975 *p* < 0.01 Reproducibility = 3.0%
[88]	2021	CRISPR/Cpf1	RNA	Synthetic dengue viral RNA in buffered solutions	100 fM	Electrochemical CRISPR reaction–based biosensor conjugating methylene blue to amplify the signal with AuNPs.	Electrochemical signal vs. DNA concentration: R^2^ = 0.9848 at 95% confidence level Detection time = ~30 min
[90]	2021	CRISPR/Cas13a	RNA	Synthetic Dengue viral RNA in buffer	0.78 fM	Electrochemical sensor with hairpin assembly on probe surface that activates a CRISPR reaction when target RNA is detected.	Linear Detection Range = 5fM–50 nM Peak current vs. DENV–1 concentration (Log): R^2^ = 0.9969
[58]	2022	SPR	NS1	Blood serum	60 ng/mL	Alkanethiol–based self–assembled monolayer for anti–NSI antibody binding on the surface of unclad fiber coated with silver.	Sensitivity at lowest concentration is 54.7 nm/(µg/mL)
[61]	2022	SPR	NS1 mAb	Simulated sensing medium	N/A	SPR properties together with gold/EDC–NHS/IgG components.	N/A
[66]	2022	Photonic Crystals	Plasma, Platelets. and Hemoglobin	Blood refractive index variation representing dengue—infected blood	9.3 × 10^−3^ RIU	1D photonic crystals made of a [(Si/LiF)^6^D(LiF/Si)^6^] layer with Defect layer being a function of plasma, platelets and hemoglobin.	Q Factor = 1569 Sensitivity = 203.09 nm/RIU
[74]	2022	EIS	NS1	Serum samples on a single chip	1.17 ng/mL	Gold surface electrodes functionalized by a self–assembled biorecognition monolayer.	Concentration range = 15.62–500 ng/mL Percent change impedance vs. NS1concentration (Log): R^2^ = 0.990
[84]	2022	SRR	Blood	Blood—mimicking samples with controlled dielectric properties	N/A	Double–layer metamaterial–based resonator with a replaceable top layer that can detect the dielectric properties of blood samples operating in the millimeter wave range.	Sensitivity = 0.325 GHz R^2^ = 0.9729 Substrate layer 1 thickness = 1.57 mm Substrate layer 2 thickness = 0.127 mm
[85]	2022	Microfluidics	RNA	Whole blood	N/A	Paper/polymer strip microfluidic biosensor utilizing nucleic acid isolation, isothermal amplification, and colorimetry.	Acquisition time of ~30 min A sensitivity and specificity of 95% and 100% were calculated for purified RNA and for blood serum, a sensitivity of 91% and a specificity of 100% was achieved.
[89]	2022	CRISPR/Cas12a	RNA	Synthetic viral DNA/RNA targets in buffer	51 fM	Fluorescent detection of DENV RNA using CRISPR–based reaction to amplify the signal by target–triggered hybridization chain reaction.	Linear Detection Range = 1pM–10 nM Fluorescence intensity vs. DENV–1 RNA concentration (Log): R^2^ = 0.9962 Recovery test range = 92.4–106.7% Relative Standard Deviation = 3.1–6.8%
[55]	2023	Colorimetry	NS1	Recombinant DENV–2 NS1–Spiked buffer and serum	1.56 ng/mL	Thermochromic sheet temperature sensor coupled with a lateral flow assay.	Detection limit improved by up to 4 times compared to normal value of 6.25 ng/mL
[59]	2023	SPR	NS1	Blood plasma, platelets, and hemoglobin	N/A	Sequential layering of the BK7 prism, silver, titanium disilicide, black phosphorus and sensing medium.	Maximum Sensitivity = 257.3 deg/RIU Quality Factor = 85.45 deg^−1^ Detection accuracy = 0.54RIU^−1^
[78]	2023	SRR	Blood	Simulated dielectric environments	N/A	Metamaterial–based biosensor with four square split–ring resonators coupled with a graphene ring with absorption in the terahertz range.	Sensitivity = 1.7 THz/RIU Figure of Merit = 165.09 RIU^−1^ Q factor = 112.5 Substrate Thickness = 3 µm
[86]	2023	Microfluidics	IgG	Serum	3.1 × 10^−4^ ng/mL	A surface–integrated microfluidic platform was fabricated using zinc oxide nanorods synthesized via a seed–assisted thermal technique.	Dynamic Detection Range = 3.1 × 10^3^–3.1 × 10^−4^ ng/mL Detection time = ~15 min

* Abbreviations: SPR—Surface plasmon resonance, LSPR—Localized Surface plasmon resonance, SERS—Surface–enhanced Raman scattering, DPV—Differential pulse voltammetry, EIS—Electrochemical impedance spectroscopy, CRISPR—Clustered regularly interspaced short palindromic repeats. N/A—Value not reported in the original study

## Data Availability

No new data were created.

## Abbreviations

The following abbreviations are used in this manuscript:

TLA	Three letter acronyms
DENV	Dengue Virus
NS1	Non–structural protein 1
PCR	Polymerase chain reaction
RT–PCR	Reverse transcriptase–polymerase chain reaction
RT–qPCR	Real–time quantitative RT–PCR
LOD	Limit of Detection
ELISA	Enzyme–linked immunosorbent assays
LFA	Lateral Flow Assays
AuNPs	Gold nanoparticles
POC	Point–of–Care
IFA	Immunofluorescence based assays
SPR	Surface plasmon resonance
LSPR	Localized Surface plasmon resonance
SERS	Surface–enhanced Raman scattering
DPV	Differential pulse voltammetry
EIS	Electrochemical impedance spectroscopy
SRR	Split ring resonator
CSRR	Complementary split ring resonator
RT–LAMP	Reverse transcriptase—loop mediated isothermal amplification
CRISPR	Clustered regularly interspaced short palindromic repeats
SHERLOCK	Specific High–sensitivity Enzymatic Reporter un–LOCKing
DETECTR	DNA Endonuclease—Targeted CRISPR Trans Reporter
AI	Artificial Intelligence
ML	Machine Learning
DL	Deep learning
CBC	Complete Blood Count
SVM	Support Vector Machine
ETC	Extra Tree Classifier
ANN	Artificial Neural Network
AUC	Area Under Curve
DNN	Deep neural network
LR	Logistic regression

## References

[1] World Health Organization Dengue and Severe Dengue. https://www.who.int/news-room/fact-sheets/detail/dengue-and-severe-dengue.

[2] Cafferata M.L., Bardach A., Rey-Ares L., Alcaraz A., Cormick G., Gibbons L., Romano M., Cesaroni S., Ruvinsky S. (2013). Dengue Epidemiology and Burden of Disease in Latin America and the Caribbean: A Systematic Review of the Literature and Meta-Analysis. Value Health Reg. Issues.

[3] Van Kleef E., Bambrick H., Hales S. (2011). The geographic distribution of dengue fever and the potential influence of global climate change. ISEE Conf. Abstr..

[4] World Health Organization Dengue—Global Situation. https://www.who.int/emergencies/disease-outbreak-news/item/2023-DON498.

[5] Haider N., Hasan M.N., Onyango J., Asaduzzaman M. (2024). Global landmark: 2023 marks the worst year for dengue cases with millions infected and thousands of deaths reported. IJID Reg..

[6] Dengue Fever Outbreak in Jeddah, Saudi Arabia: September 2022 to April 2023|Field Epidemiology Training Program. https://www.saudifetp.org/seb/dengue-fever-outbreak-jeddah-saudi-arabia-september-2022-april-2023.

[7] Alyahya H.S. (2023). Prevalence of dengue fever in Saudi Arabia: Jeddah as a case study. Entomol. Res..

[8] Dengue Fever Awareness Starts in Jeddah|National Platform (National Portal). https://my.gov.sa/en/news/3126.

[9] Laporta G.Z., Potter A.M., Oliveira J.F.A., Bourke B.P., Pecor D.B., Linton Y.-M. (2023). Global Distribution of Aedes aegypti and Aedes albopictus in a Climate Change Scenario of Regional Rivalry. Insects.

[10] Clark G.G., Suárez M.F. (1992). Mosquito vector control and biology in Latin America—A second symposium. J. Am. Mosq. Control Assoc..

[11] Thisyakorn U., Thisyakorn C. (2013). Latest developments and future directions in dengue vaccines. Ther. Adv. Vaccines.

[12] Bhattacharya M.K., Maitra S., Ganguly A., Bhattacharya A., Sinha A. (2013). Dengue: A Growing Menace—A Snapshot of Recent Facts, Figures & Remedies. Int. J. Biomed. Sci..

[13] Smith C.E.G. (1956). The History of Dengue in Tropical Asia and its Probable Relationship to the Mosquito *Aedes aegypti*. J. Trop. Med. Hyg..

[14] Mustafa M., Rasotgi V., Jain S., Gupta V. (2015). Discovery of fifth serotype of dengue virus (DENV-5): A new public health dilemma in dengue control. Med. J. Armed Forces India.

[15] Marchette N.J., Rudnick A., Garcia R., MacVean D.W. (1978). Alphaviruses in Peninusular Malaysia: I. Virus isolations and animal serology. Southeast Asian J. Trop. Med. Public Health.

[16] Robert V., Lhuillier M., Meunier D., Sarthou J.L., Monteny N., Digoutte J.P., Cornet M., Germain M., Cordellier R. (1993). Yellow fever virus, dengue 2 and other arboviruses isolated from mosquitos, in Burkina Faso, from 1983 to 1986. Entomological and epidemiological considerations. Bull. Soc. Pathol. Exot..

[17] Ramage H., Cherry S. (2015). Virus-Host Interactions: From Unbiased Genetic Screens to Function. Annu. Rev. Virol..

[18] Bessaud M., Pastorino B.A.M., Peyrefitte C.N., Rolland D., Grandadam M., Tolou H.J. (2006). Functional characterization of the NS2B/NS3 protease complex from seven viruses belonging to different groups inside the genus *Flavivirus*. Virus Res..

[19] Islam M.T., Quispe C., Herrera-Bravo J., Sarkar C., Sharma R., Garg N., Fredes L.I., Martorell M., Alshehri M.M., Sharifi-Rad J. (2021). Production, Transmission, Pathogenesis, and Control of Dengue Virus: A Literature-Based Undivided Perspective. BioMed Res. Int..

[20] Ashley E.A. (2011). Dengue fever. Trends Anaesth. Crit. Care.

[21] Darwish N.T., Alias Y.B., Khor S.M. (2015). An introduction to dengue-disease diagnostics. TrAC Trends Anal. Chem..

[22] Pervin M., Shahina T., Sil B.K., Md N.I. Isolation and Serotyping of Dengue Viruses by Mosquito Inoculation and Cell Culture Technique: An Experience in Bangladesh. World Health Organization. https://iris.who.int/handle/10665/163882.

[23] Choy M.M., Gubler D.J. (2014). Isolation and titration of dengue viruses by the mosquito inoculation technique. Methods Mol. Biol..

[24] Vene S., Mangiafico J., Niklasson B. (1995). Indirect immunofluorescence for serological diagnosis of dengue virus infections in Swedish patients. Clin. Diagn. Virol..

[25] Martinez-Liu C., Machain-Williams C., Martinez-Acuña N., Lozano-Sepulveda S., Galan-Huerta K., Arellanos-Soto D., Meléndez-Villanueva M., Ávalos-Nolazco D., Pérez-Ibarra K., Galindo-Rodríguez S. (2022). Development of a Rapid Gold Nanoparticle-Based Lateral Flow Immunoassay for the Detection of Dengue Virus. Biosensors.

[26] Jalali M., Zaborowska J., Jalali M., Jalali M., Saldanha F.Y.L., Jalali M. (2017). Chapter 1—The Polymerase Chain Reaction: PCR, qPCR, and RT-PCR. Basic Science Methods for Clinical Researchers.

[27] Harris E., Roberts T.G., Smith L., Selle J., Kramer L.D., Valle S., Sandoval E., Balmaseda A. (1998). Typing of Dengue Viruses in Clinical Specimens and Mosquitoes by Single-Tube Multiplex Reverse Transcriptase PCR. J. Clin. Microbiol..

[28] Miller T.E., Beltran W.F.G., Bard A.Z., Gogakos T., Anahtar M.N., Astudillo M.G., Yang D., Thierauf J., Fisch A.S., Mahowald G.K. (2020). Clinical sensitivity and interpretation of PCR and serological COVID-19 diagnostics for patients presenting to the hospital. FASEB J..

[29] (2009). Laboratory Diagnosis And Diagnostic Tests. Dengue: Guidelines for Diagnosis, Treatment, Prevention and Control: New Edition.

[30] Jiang K., Lee J.-H., Fung T.S., Wu J., Liu C., Mi H., Rajapakse R.J., Balasuriya U.B., Peng Y.-K., Go Y.Y. (2023). Next-generation diagnostic test for dengue virus detection using an ultrafast plasmonic colorimetric RT-PCR strategy. Anal. Chim. Acta.

[31] Kabir A., Zilouchian H., Younas M.A., Asghar W. (2021). Dengue Detection: Advances in Diagnostic Tools from Conventional Technology to Point of Care. Biosensors.

[32] Musso D., Desprès P. (2020). Serological Diagnosis of Flavivirus-Associated Human Infections. Diagnostics.

[33] Darwish N.T., Sekaran S.D., Alias Y., Khor S.M. (2018). Immunofluorescence–based biosensor for the determination of dengue virus NS1 in clinical samples. J. Pharm. Biomed. Anal..

[34] Chen P.-K., Chang J.-H., Ke L.-Y., Kao J.-K., Chen C.-H., Yang R.-C., Yoshimura T., Ito E., Tsai J.-J. (2023). Advanced Detection Method for Dengue NS1 Protein Using Ultrasensitive ELISA with Thio-NAD Cycling. Viruses.

[35] Watabe S., Kodama H., Kaneda M., Morikawa M., Nakaishi K., Yoshimura T., Iwai A., Miura T., Ito E. (2014). Ultrasensitive enzyme-linked immunosorbent assay (ELISA) of proteins by combination with the thio-NAD cycling method. Biophysics.

[36] Ding S.C. (2008). Virology: Principles and Applications. Yale J. Biol. Med..

[37] Dengue Guidelines, for Diagnosis, Treatment, Prevention and Control. https://www.who.int/publications/i/item/9789241547871.

[38] Jarman R.G., Nisalak A., Anderson K.B., Klungthong C., Thaisomboonsuk B., Kaneechit W., Kalayanarooj S., Gibbons R.V. (2011). Factors influencing dengue virus isolation by C6/36 cell culture and mosquito inoculation of nested PCR-positive clinical samples. Am. J. Trop. Med. Hyg..

[39] Scarpaleggia M., Garzillo G., Lucente M., Fraccalvieri C., Randazzo N., Massaro E., Galano B., Ricucci V., Bruzzone B., Domnich A. (2024). Diagnostic Accuracy of Five Molecular Assays for the Detection of Dengue Virus. Medicina.

[40] Christenson R.H., Snyder S.R., Shaw C.S., Derzon J.H., Black R.S., Mass D., Epner P., Favoretto A.M., Liebow E.B. (2011). Laboratory Medicine Best Practices: Systematic Evidence Review and Evaluation Methods for Quality Improvement. Clin. Chem..

[41] (2025). Emerging Technologies in Point-of-Care Molecular Diagnostics for Resource-Limited Settings|Request PDF. ResearchGate. https://www.researchgate.net/publication/262015193_Emerging_technologies_in_point-of-care_molecular_diagnostics_for_resource-limited_settings.

[42] CDC Serologic Tests for Dengue Virus. Dengue. https://www.cdc.gov/dengue/hcp/diagnosis-testing/serologic-tests-for-dengue-virus.html.

[43] Lavanya N., Fazio E., Neri F., Bonavita A., Leonardi S.G., Neri G., Sekar C. (2015). Simultaneous electrochemical determination of epinephrine and uric acid in the presence of ascorbic acid using SnO_2_/graphene nanocomposite modified glassy carbon electrode. Sens. Actuators B Chem..

[44] Gaspar-Castillo C., Rodríguez M.H., Ortiz-Navarrete V., Alpuche-Aranda C.M., Martinez-Barnetche J. (2023). Structural and immunological basis of cross-reactivity between dengue and Zika infections: Implications in serosurveillance in endemic regions. Front. Microbiol..

[45] Haleem A., Javaid M., Singh R.P., Suman R., Rab S. (2021). Biosensors applications in medical field: A brief review. Sensors Int..

[46] Akgönüllü S., Denizli A. (2022). Recent advances in optical biosensing approaches for biomarkers detection. Biosens. Bioelectron. X.

[47] Rasheed S., Kanwal T., Ahmad N., Fatima B., Najam-Ul-Haq M., Hussain D. (2024). Advances and challenges in portable optical biosensors for onsite detection and point-of-care diagnostics. TrAC Trends Anal. Chem..

[48] Shanbhag M.M., Manasa G., Mascarenhas R.J., Mondal K., Shetti N.P. (2023). Fundamentals of bio-electrochemical sensing. Chem. Eng. J. Adv..

[49] Zhao J.-M., Wang Y.-K., Shi B.-W., Wang Y.-X., Jiang Y.-F., Yang G.-L., Gao X.-D., Qiang T. (2024). Microwave biosensor for the detection of growth inhibition of human liver cancer cells at different concentrations of chemotherapeutic drug. Front. Bioeng. Biotechnol..

[50] Liu Z., Zhou Y., Lu J., Gong T., Ibáñez E., Cifuentes A., Lu W. (2024). Microfluidic biosensors for biomarker detection in body fluids: A key approach for early cancer diagnosis. Biomark. Res..

[51] Bakhtiari A., Kähler C.J. (2024). A method to prevent clogging and clustering in microfluidic systems using microbubble streaming. Biomicrofluidics.

[52] Liberty J.T., Bromage S., Peter E., Ihedioha O.C., Alsalman F.B., Odogwu T.S. (2025). CRISPR revolution: Unleashing precision pathogen detection to safeguard public health and food safety. Methods.

[53] What Are the Ethical Concerns of Genome Editing?. https://www.genome.gov/about-genomics/policy-issues/Genome-Editing/ethical-concerns.

[54] Damborský P., Švitel J., Katrlík J. (2016). Optical biosensors. Essays Biochem..

[55] Trakoolwilaiwan T., Takeuchi Y., Leung T.S., Sebek M., Storozhuk L., Nguyen L., Tung L.D., Thanh N.T.K. (2023). Development of a thermochromic lateral flow assay to improve sensitivity for dengue virus serotype 2 NS1 detection. Nanoscale.

[56] Perumal V., Hashim U. (2014). Advances in biosensors: Principle, architecture and applications. J. Appl. Biomed..

[57] SariSabban (2011). English: Surface Plasmon Resonance (SPR) Configuration. https://commons.wikimedia.org/w/index.php?curid=18139885.

[58] Gahlaut S.K., Pathak A., Gupta B.D., Singh J. (2022). Portable fiber-optic SPR platform for the detection of NS1-antigen for dengue diagnosis. Biosens. Bioelectron..

[59] Almawgani A.H.M., Sarkar P., Pal A., Srivastava G., Uniyal A., Alhawari A.R.H., Muduli A. (2023). Titanium Disilicide, Black Phosphorus–Based Surface Plasmon Resonance Sensor for Dengue Detection. Plasmonics.

[60] Versiani A.F., Martins E.M.N., Andrade L.M., Cox L., Pereira G.C., Barbosa-Stancioli E.F., Nogueira M.L., Ladeira L.O., da Fonseca F.G. (2020). Nanosensors based on LSPR are able to serologically differentiate dengue from Zika infections. Sci. Rep..

[61] Aprilia L., Mayasari R.D., Yanza N., Pambudi S., Tarwadi, Budi A.S., Nuryadi R. (2022). Surface Plasmon Resonance Sensor Based on Kretschmann Configuration for Dengue Virus Detection. Proceedings of the 2022 International Conference on Radar, Antenna, Microwave, Electronics, and Telecommunications (ICRAMET).

[62] Gahlaut S.K., Savargaonkar D., Sharan C., Yadav S., Mishra P., Singh J.P. (2020). SERS Platform for Dengue Diagnosis from Clinical Samples Employing a Hand Held Raman Spectrometer. Anal. Chem..

[63] Sánchez-Purrà M., Carré-Camps M., de Puig H., Bosch I., Gehrke L., Hamad-Schifferli K. (2017). Surface-Enhanced Raman Spectroscopy-Based Sandwich Immunoassays for Multiplexed Detection of Zika and Dengue Viral Biomarkers. ACS Infect. Dis..

[64] Li T., Liu G., Kong H., Yang G., Wei G., Zhou X. (2022). Recent advances in photonic crystal-based sensors. Coord. Chem. Rev..

[65] Dakhlaoui H., Belhadj W., Elabidi H., Al-Shameri N.S., Ungan F., Wong B.M. (2024). Harnessing a Dielectric/Plasma Photonic Crystal as an Optical Microwave Filter: Role of Defect Layers and External Magnetic Fields. Materials.

[66] Sharma S., Kumar A. (2021). Design of a Biosensor for the Detection of Dengue Virus Using 1D Photonic Crystals. Plasmonics.

[67] Butt M., Khonina S., Kazanskiy N. (2021). Recent advances in photonic crystal optical devices: A review. Opt. Laser Technol..

[68] Grieshaber D., MacKenzie R., Vörös J., Reimhult E. (2008). Electrochemical Biosensors—Sensor Principles and Architectures. Sensors.

[69] Mendonça P.D., Santos L.K.B., Foguel M.V., Rodrigues M.A.B., Cordeiro M.T., Gonçalves L.M., Marques E.T.A., Dutra R.F. (2021). NS1 glycoprotein detection in serum and urine as an electrochemical screening immunosensor for dengue and Zika virus. Anal. Bioanal. Chem..

[70] Chowdhury A.D., Ganganboina A.B., Nasrin F., Takemura K., Doong R.-A., Utomo D.I.S., Lee J., Khoris I.M., Park E.Y. (2018). Femtomolar Detection of Dengue Virus DNA with Serotype Identification Ability. Anal. Chem..

[71] Kanagavalli P., Veerapandian M. (2020). Opto-electrochemical functionality of Ru(II)-reinforced graphene oxide nanosheets for immunosensing of dengue virus non-structural 1 protein. Biosens. Bioelectron..

[72] Abu Odeh A., Al-Douri Y., Voon C.H., Ayub R.M., Gopinath S.C.B., Abu Odeh R., Ameri M., Bouhemadou A. (2017). A needle-like Cu2CdSnS4 alloy nanostructure-based integrated electrochemical biosensor for detecting the DNA of Dengue serotype 2. Microchim. Acta.

[73] Lazanas A.C., Prodromidis M.I. (2023). Electrochemical Impedance Spectroscopy─A Tutorial. ACS Meas. Sci. Au.

[74] Sampaio I., Quatroni F.D., Costa J.N.Y., Zucolotto V. (2022). Electrochemical detection of Zika and Dengue infections using a single chip. Biosens. Bioelectron..

[75] Dutta R., Thangapandi K., Mondal S., Nanda A., Bose S., Sanyal S., Jana S.K., Ghorai S. (2020). Polyaniline Based Electrochemical Sensor for the Detection of Dengue Virus Infection. Avicenna J. Med. Biotechnol..

[76] RoyChoudhury S., Rawat V., Jalal A.H., Kale S., Bhansali S. (2016). Recent advances in metamaterial split-ring-resonator circuits as biosensors and therapeutic agents. Biosens. Bioelectron..

[77] Salim A., Lim S. (2018). Review of Recent Metamaterial Microfluidic Sensors. Sensors.

[78] Upender P., Bharathi S.P., Sukriti, Kumba K., Kumar A. (2023). A Compact Metamaterial Biosensor for Multi-Virus Detection with Tunability and High Incidence Angle Absorption. IEEE Access.

[79] Kothai R., Arul B., Kothai R., Arul B. (2020). Dengue Fever: An Overview. Dengue Fever in a One Health Perspective.

[80] Banerjee A., Tripathi A., Duggal S., Banerjee A., Vrati S. (2020). Dengue virus infection impedes megakaryopoiesis in MEG-01 cells where the virus envelope protein interacts with the transcription factor TAL-1. Sci. Rep..

[81] CDC Pathophysiology of Dengue Virus Infection, Centers for Disease Control and Prevention. https://www.cdc.gov/dengue/training/cme/ccm/Pathophysiology.pdf.

[82] Hematocrit Test: What It Is, Levels, High & Low Range. Cleveland Clinic. https://my.clevelandclinic.org/health/diagnostics/17683-hematocrit.

[83] Ralapanawa U., Alawattegama A.T.M., Gunrathne M., Tennakoon S., Kularatne S.A.M., Jayalath T. (2018). Value of peripheral blood count for dengue severity prediction. BMC Res. Notes.

[84] Qureshi S.A., Abidin Z.Z., Majid H.A., Ashyap A.Y., See C.H. (2022). Double-Layered metamaterial resonator operating at millimetre wave for detection of dengue virus. AEU—Int. J. Electron. Commun..

[85] Biswas P., Sulochana G.N.M., Banuprasad T.N., Goyal P., Modak D., Ghosh A.K., Chakraborty S. (2022). All-Serotype Dengue Virus Detection through Multilayered Origami-Based Paper/Polymer Microfluidics. ACS Sens..

[86] Pormrungruang P., Phanthanawiboon S., Jessadaluk S., Larpthavee P., Thaosing J., Rangkasikorn A., Kayunkid N., Waiwijit U., Horprathum M., Klamchuen A. (2023). Metal Oxide Nanostructures Enhanced Microfluidic Platform for Efficient and Sensitive Immunofluorescence Detection of Dengue Virus. Nanomaterials.

[87] Kim S., Ji S., Koh H.R. (2021). CRISPR as a Diagnostic Tool. Biomolecules.

[88] Lee Y., Choi J., Han H.K., Park S., Park S.Y., Park C., Bark C., Lee T., Min J. (2021). Fabrication of ultrasensitive electrochemical biosensor for dengue fever viral RNA Based on CRISPR/Cpf1 reaction. Sens. Actuators B Chem..

[89] Zhong M., Liu J., Wu J., Li J., Luo N., Zhu C., Liu R., Xia Q., Ju H. (2022). Proximity sequence enhanced CRISPR-Cas12a connected through hybridization chain reaction for sensitive biosensing of dengue virus. Sens. Actuators B Chem..

[90] Wang J., Xia Q., Wu J., Lin Y., Ju H. (2021). A sensitive electrochemical method for rapid detection of dengue virus by CRISPR/Cas13a-assisted catalytic hairpin assembly. Anal. Chim. Acta.

[91] What Is a Colorimeter? Definition, Working & Key Advantages. https://www.prestogroup.com/blog/what-is-a-colorimeter-definition-working-advantages/.

[92] Fundamentals of Colorimetry|IntechOpen. https://www.intechopen.com/chapters/87730.

[93] Wei J. 6 Advantages of Surface Plasmon Resonance Technology. Affinite Instruments. https://www.affiniteinstruments.com/post/6-advantages-of-surface-plasmon-resonance-technology.

[94] Mallika C.S., Shwetha M. (2025). Plasmonic Ring Resonator-Based Sensors: Design, Performance, and Applications. Plasmonics.

[95] Han X.X., Rodriguez R.S., Haynes C.L., Ozaki Y., Zhao B. (2021). Surface-enhanced Raman spectroscopy. Nat. Rev. Methods Prim..

[96] Li M., Lai X., Li C., Song Y. (2019). Recent advantages of colloidal photonic crystals and their applications for lumnescence enhancement. Mater. Today Nano.

[97] Firouzjaei A.S., Afghahi S.S., Valmoozi A.-A.E., Firouzjaei A.S., Afghahi S.S., Valmoozi A.-A.E. (2024). Emerging Trends, Applications, and Fabrication Techniques in Photonic Crystal Technology. Recent Advances and Trends in Photonic Crystal Technology.

[98] Kashyap B., Kumar R. (2022). A novel multi-set differential pulse voltammetry technique for improving precision in electrochemical sensing. Biosens. Bioelectron..

[99] Mota F.A., Passos M.L., Santos J.L., Saraiva M.M. (2024). Comparative analysis of electrochemical and optical sensors for detection of chronic wounds biomarkers: A review. Biosens. Bioelectron..

[100] Strong M.E., Richards J.R., Torres M., Beck C.M., La Belle J.T. (2021). Faradaic electrochemical impedance spectroscopy for enhanced analyte detection in diagnostics. Biosens. Bioelectron..

[101] Microfluidic Point-of-Care (POC) Devices in Early Diagnosis: A Review of Opportunities and Challenges—PMC. https://pmc.ncbi.nlm.nih.gov/articles/PMC8875995/.

[102] Vealan K., Joseph N., Alimat S., Karumbati A.S., Thilakavathy K. (2023). Lateral flow assay: A promising rapid point-of-care testing tool for infections and non-communicable diseases. Asian Biomed..

[103] Riya N.J., Chakraborty M., Khan R. (2024). Artificial Intelligence-Based Early Detection of Dengue Using CBC Data. IEEE Access.

[104] Qaiser A., Manzoor S., Hashmi A.H., Javed H., Zafar A., Ashraf J. (2024). Support Vector Machine Outperforms Other Machine Learning Models in Early Diagnosis of Dengue Using Routine Clinical Data. Adv. Virol..

[105] Abdualgalil B., Abraham S., Ismael W.M. (2022). Early Diagnosis for Dengue Disease Prediction Using Efficient Machine Learning Techniques Based on Clinical Data. J. Robot. Control (JRC).

[106] Iqbal N., Islam M. (2019). Machine learning for dengue outbreak prediction: A performance evaluation of different prominent classifiers. Informatica.

[107] Exebio-Chepe Y.V., Bravo-Ruiz J.A., Tuesta-Montesa V.A. (2024). Comparison of machine learning algorithms for dengue virus (DENV) classification. J. Appl. Res. Technol..

[108] Ho T.-S., Weng T.-C., Wang J.-D., Han H.-C., Cheng H.-C., Yang C.-C., Yu C.-H., Liu Y.-J., Hu C.H., Huang C.-Y. (2020). Comparing machine learning with case-control models to identify confirmed dengue cases. PLoS Neglected Trop. Dis..

[109] Ansari S., Jain D., Budhiraja S. (2023). Machine-learning prediction models for any blood component transfusion in hospitalized dengue patients. Hematol. Transfus. Cell Ther..

[110] Hassan M., Ali S., Saleem M., Sanaullah M., Fahad L.G., Kim J.Y., Alquhayz H., Tahir S.F. (2022). Diagnosis of dengue virus infection using spectroscopic images and deep learning. PeerJ Comput. Sci..

[111] Santos C.Y., Tuboi S., Abreu A.d.J.L.d., Abud D.A., Neto A.A.L., Pereira R., Siqueira J.B. (2023). A machine learning model to assess potential misdiagnosed dengue hospitalization. Heliyon.

[112] All Imputation Techniques with Pros and Cons. https://kaggle.com/code/azminetoushikwasi/all-imputation-techniques-with-pros-and-cons.

[113] ReplaceMissingValues. https://weka.sourceforge.io/doc.dev/weka/filters/unsupervised/attribute/ReplaceMissingValues.html.

[114] Zhou M., Li J., Yuan S., Yang X., Lu J., Jiang B. (2023). A centrifugal microfluidic system for automated detection of multiple heavy metal ions by aptamer-based colorimetric assay. Sens. Actuators B Chem..

